# Transcriptome analysis reveals a comprehensive insect resistance response mechanism in cotton to infestation by the phloem feeding insect *Bemisia tabaci* (whitefly)

**DOI:** 10.1111/pbi.12554

**Published:** 2016-03-31

**Authors:** Jianying Li, Lizhen Zhu, J. Joe Hull, Sijia Liang, Henry Daniell, Shuangxia Jin, Xianlong Zhang

**Affiliations:** ^1^ National Key Laboratory of Crop Genetic Improvement Huazhong Agricultural University Wuhan Hubei China; ^2^ USDA‐ARS Arid Land Agricultural Research Center Maricopa AZ USA; ^3^ Department of Biochemistry School of Dental Medicine University of Pennsylvania Philadelphia PA USA

**Keywords:** cotton, whitefly, RNA‐Seq, DEGs, co‐expression, transcription factor

## Abstract

The whitefly (*Bemisia tabaci*) causes tremendous damage to cotton production worldwide. However, very limited information is available about how plants perceive and defend themselves from this destructive pest. In this study, the transcriptomic differences between two cotton cultivars that exhibit either strong resistance (HR) or sensitivity (ZS) to whitefly were compared at different time points (0, 12, 24 and 48 h after infection) using RNA‐Seq. Approximately one billion paired‐end reads were obtained by Illumina sequencing technology. Gene ontology and KEGG pathway analysis indicated that the cotton transcriptional response to whitefly infestation involves genes encoding protein kinases, transcription factors, metabolite synthesis, and phytohormone signalling. Furthermore, a weighted gene co‐expression network constructed from RNA‐Seq datasets showed that *WRKY40* and copper transport protein are hub genes that may regulate cotton defenses to whitefly infestation. Silencing *GhMPK3* by virus‐induced gene silencing (VIGS) resulted in suppression of the MPK‐WRKY‐JA and ET pathways and lead to enhanced whitefly susceptibility, suggesting that the candidate insect resistant genes identified in this RNA‐Seq analysis are credible and offer significant utility. Taken together, this study provides comprehensive insights into the cotton defense system to whitefly infestation and has identified several candidate genes for control of phloem‐feeding pests.

## Introduction

While cotton is a fibre and oil‐yielding crop of great economic importance, productivity is severely affected by both biotic and abiotic stresses (Razaq *et al*., 2004). Currently, cotton is one of the four major transgenic crops in the world and the land area devoted to its cultivation has been steadily increasing over the last decade. With the widespread popularization of *Bt* cotton, lepidopteran pests such as *Helicoverpa armigera* and *Pectinophora gossypiella* have been successfully controlled (Li *et al*., 2014; Lu *et al*., 2010). However, *Bt* toxins are ineffectual against phloem‐feeding pests, such as whitefly, aphid and leafhopper. The highly specialized mode of feeding by these pests present a unique stress on the host plant (Kempema *et al*., 2007).

Whiteflies (*Bemisia tabaci*) are genetically diverse cryptic species complex consisting of ~30 morphologically indistinguishable species (De Barro *et al*., 2011; Dinsdale *et al*., 2010; Hu *et al*., 2011) that are pests of more than 500 plant species including many economically important crops such as cotton. Whiteflies negatively impact crop production through direct feeding, induction of host plant phytotoxic disorders, transmission of diverse viruses, and honeydew‐associated fungal growths (Inbar and Gerling, 2008; Jones, 2003). With prolonged infestation, they will cause shedding of young cotton bolls, resulting in considerable cotton fibre yield loss. Among the whitefly complex, species commonly referred to as B‐ and Q‐biotypes are the most invasive and economically damaging throughout the world. In China, the more destructive B‐biotype, also referred to as the Middle East‐Asia Minor 1 (MEAM1) species, was initially detected in the mid 1990s (Tay *et al*., 2012). In contrast, the Q‐type, classified as the Mediterranean (MED) species, is a more recent introduction into China (Chu *et al*., 2006). However, both species have rapidly extended throughout the country (Zhang *et al*., 2005). Because whiteflies are capable of long, continuous interactions with the plant host (Kempema *et al*., 2007), they are an ideal model system for studying the interactions between phloem‐feeding pests and the plant host.

Plants are frequently attacked by both insects and pathogens. They have therefore evolved elaborate defense systems during the ~350 million years of co‐evolution with plants (Gatehouse, 2002; Mithöfer and Boland, 2008). In addition to the physical barrier (i.e. thickness and trichomes), secondary metabolites and toxic compounds, plants have also evolved a constitutive defense system. For direct defense, plants mobilize a variety of defense signalling pathways and activate the expression of defense genes that function in the synthesis of secondary metabolites. Compelling evidence has accumulated indicating the crucial importance of plant secondary metabolites, such as gossypol, glucosinolates, cyanogenic glucosides, alkaloids, phenolics, and proteinase inhibitors (PIs), functioning as toxins, repellents and/or anti‐digestives (Arimura *et al*., 2011; Fürstenberg‐Hägg *et al*., 2013; Wu and Baldwin, 2010). The plant defense system is regulated by a suite of phytohormones, including jasmonic acid (JA), salicylic acid (SA), ethylene (ET), abscisic acid (ABA), brassinosteroids (BR), and gibberellin (GA; Arimura *et al*., 2005; Jin *et al*., 2011; Kempema *et al*., 2007; Loake and Grant, 2007; Sun *et al*., 2011). In the indirect defense system, plants release volatile substances that attract natural enemies of the pest, thus enlisting the aid of another organism to reduce damage to the plant. Evolution of these two powerful defense systems in plants have enabled them to effectively compete in their evolutionary arms race with herbivores (Kessler and Baldwin, 2002).

The initial step in activation of the plant response is recognition of pathogen or herbivory‐induced damage. Various microbial (or pathogen)‐associated molecular patterns (MAMPs or PAMPs) are recognized by specific receptors (Mithöfer and Boland, 2008). Herbivory‐associated interaction molecular patterns (HAMPs) in particular may activate pattern recognition receptors (PRRs) and PAMP‐triggered immunity (PTI; Boller and Felix, 2009). Damage‐associated molecular patterns (DAMPs) are endogenous molecular mechanisms produced by the plant hosts after infection and are also recognized by PRRs to trigger defensive reactions (Jones and Dangl, 2006). All these herbivory‐induced changes are mediated by elaborate signalling networks, which include receptors/sensors, Ca^2+^ influx pathways, kinase cascades, the formation of reactive oxygen species (ROS), secondary metabolites, mitogen‐activated protein kinases (MAPKs), calcium‐dependent protein kinases (CDPKs), and phytohormone signalling pathways (Lin *et al*., 2015; Wu and Baldwin, 2010).

For sap‐sucking insect/plant host interactions, the rice/brown planthopper (BPH), aphid, and whitefly/Arabidopsis offer opportunities as potential models. Studies examining the transcriptional effects of BPH feeding in rice are particularly intriguing as BPH feeding induces a Ca^2+^ pathway in rice that triggers the accumulation of H_2_O_2_ and callose as well as protein transport congestion in target tissues (Hao *et al*., 2008). Since that study, 28 BPH resistant loci have been identified from cultivated and wild rice species. Among these loci are *Bph14*, a rice gene that encodes a member of the nucleotide‐binding (NB), leucine‐rich repeat disease resistance protein (LRR) immune receptor family (Cheng *et al*., 2013), and a cluster of three genes encoding plasma membrane ‐localized lectin receptor kinases (OsLecRK1‐3) that confer broad‐spectrum resistance in rice (Cheng *et al*., 2013; Liu *et al*., 2014).

Despite these advances, the comprehensive molecular mechanisms underlying plant and sap‐sucking pest interactions remain poorly defined. DNA microarray technologies have provided a high‐throughput method for studying these molecular interactions with several genes involved in cell wall modulation, ROS generation, phytohormones, and metabolic synthesis identified (Alves‐Ferreira, 2014; Artico *et al*., 2014; Kempema *et al*., 2007; Wang *et al*., 2014). Although RNA‐Seq has been used to analyse the expression profiles of stress response genes in model plants, reports describing insect/cotton interactions are limited. Transcriptome analysis of cotton flower buds infested with the cotton boll weevil highlighted the diversity of genes and pathways regulated by pest infestation, such as kinase cascades, transcription factors (TFs), and phytohormone‐signalling pathways (Artico *et al*., 2014). Another relatively recent transcriptomic study reported significant changes in the expression of transcripts associated with sugar and amino acid metabolism in cotton following aphid and whitefly infestation (Dubey *et al*., 2013).

In this study, more than 400 elite cotton cultivars (*Gossypium hirsutum*) from the major cotton growing regions in China were screened for whitefly resistance under greenhouse and field conditions. Among these cultivars, two were identified that exhibited divergent whitefly susceptibilities, one that was largely resistant (termed HR) and another that was highly susceptible (termed ZS). The transcriptomic differences between the HR and ZS cultivars at different time points (0, 12, 24 and 48 h after infestation) were assessed by RNA‐Seq (Figure S1) and several promising candidate insect‐resistant genes were tested by VIGS. The resulting phenotypes suggest that RNA‐Seq technology has significant potential to facilitate identification of functionally critical genes.

## Result

### Identification of cotton cultivars exhibiting greatest resistance (HR) or susceptibility (ZS) to whitefly infestation

**Figure 1:**
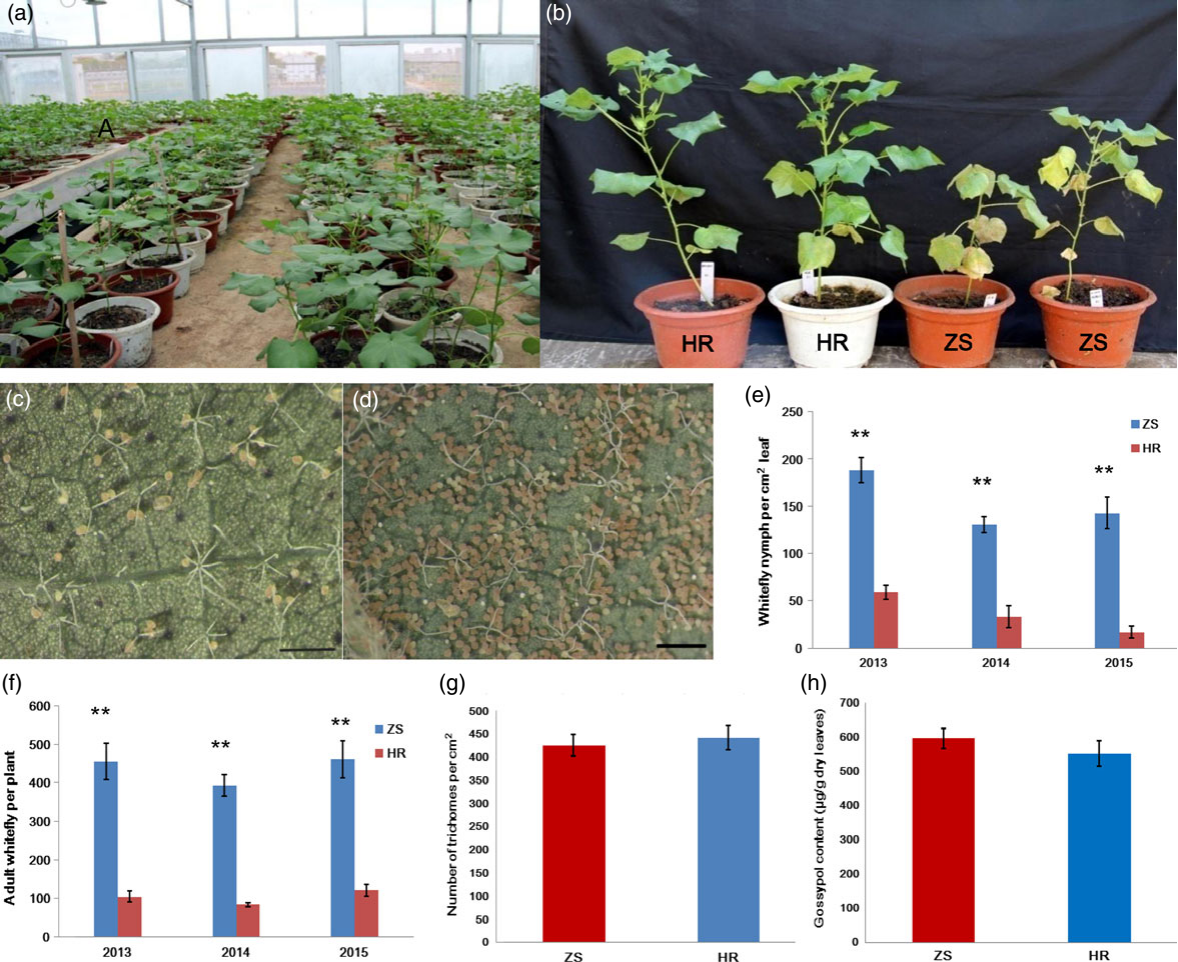
Comparison of whitefly resistance in resistant (HR) and susceptible (ZS) cotton cultivars. (a) Greenhouse‐based screen of cotton resistance to whitefly infestation. (b) Representative images of the HR and ZS cultivars following whitefly infestation. (c‐d) Representative images of immature whitefly populations (eggs and pupa) on single leaves from either the HR or ZS cultivar. Plants were infested with whitefly in the greenhouse for 8 weeks. Quantification of immature (e) and mature whitefly. (f) population densities on HR and ZS plants infested with whitefly for 8 weeks in the greenhouse. (g) Stereomicroscope‐based quantification of trichome density on the surface of HR and ZS mature leaves. (h) HPLC‐based quantification of gossypol content in the mature leaves of HR and ZS plants.

A 3‐years whitefly bioassay was carried out using multiple cotton cultivars under greenhouse conditions (Figure 1a). From more than 400 tested *G. hirsutum* cultivars, two were identified that exhibited either high levels of resistance (HR cultivar) or susceptibility (ZS cultivar) to whitefly infestation. Other than this whitefly effect, the phenotypes of the two cultivars were typical of *G. hirsutum*. We observed that the HR cultivar exhibited strong resistance to whitefly infestation and was not visibly damaged, whereas whitefly infestation caused obvious damage in the susceptible ZS cultivar (Figure 1b). Notably, the population of immature whiteflies (eggs and nymphs) on ZS was 3.3–8.5 times higher than on HR (Figure 1c–e). Adult whitefly populations were likewise significantly higher on ZS compared with HR following infestation for 1 month (Figure 1f). This trend was constant over the 3‐year experimental period. Trichomes are an important physical barrier and are usually regarded as an insect‐resistance trait utilized by cotton plants against whitefly infestation. To determine if the observed whitefly resistance phenotype was associated with trichome density, we quantified trichomes on the leaves of ZS and HR. Surprisingly, both cultivars had ~ 400 trichomes/cm^2^ leaf (Figure 1g). In addition to the trichome physical barrier, *Gossypium* species also exploit the toxicity of gossypol, a major secondary metabolism product, as protection against herbivore infestation. High Performance Liquid Chromatography (HPLC)‐based analyses, however, revealed that mature leaves of both the HR and ZS cultivars had similar gossypol content (Figure 1h). These data suggest that the phenotypic differences in whitefly susceptibility between the ZS and HR cultivars may be more mechanism‐based. Consequently, the two cultivars are ideal candidates for studying the transcriptional effects of whitefly infestation on cotton.

### Transcriptome profile of 22 RNA libraries from cotton plants infested with whiteflies at different time points

To assess the global transcriptome profile of cotton in response to a phloem feeding insect (i.e. whitefly infestation), we performed deep RNA‐Seq sequencing of the HR and ZS cultivars following whitefly infestation for 0, 12, 24, and 48 h. Three biological replicates were included at each time points for both ZS (ZS0, 12, 24, 48; ZS12 refers to the RNA library for cotton plant infested by whiteflies for 12 h) and HR cultivars. In total 24 libraries were constructed, however, two libraries were discarded due to poor data quality. A total of ~1 billion paired‐end (PE) reads were obtained from these 22 libraries with ~40–55 million reads generated per library. GC content and sequence duplication of the raw reads were calculated by FastQC software (Table 1). In total ~1 billion raw reads were obtained with ~10% of the total pair‐end reads filtered and trimmed (Table 1). Approximately 90% of the clean reads mapped to 70 478 genes in the reference *G. hirsutum* genomes (Zhang *et al*., 2015). The total mapped reads, uniquely mapped reads, and multiply mapped read are summarized in Table 1.

**Table 1:** Summary of RNA sequencing and mapping using the *Gossypium hirsutum* genome as the reference. Columns represent: number of raw sequencing reads, number of clean reads, percent of reads filtered, GC content, percent of duplicated levels, and ratio of sequences mapped to the genome. HR and ZS correspond to resistant and susceptible cultivars of upland cotton, respectively. The numerical values 1, 2, 3 indicate the different biological replicates, whereas 0, 12, 24, 48 indicate hrs of whitefly infestation. The ZS12 and ZS48 datasets consisted of two replicates, all other datasets correspond to three replicates

Sample	Total reads	Clean reads	Filter (%)	GC (%)	Dup (%)	Total mapped (%)	Uniquely mapped (%)	Multiple mapped (%)
HR0‐1	45 352 086	39 862 040	12.11	46.00	62.79	91.20	16.46	74.74
HR0‐2	49 974 678	43 839 121	12.28	46.00	68.60	93.90	16.67	77.23
HR0‐3	38 733 664	35 647 589	7.97	44.00	57.38	95.70	16.43	79.27
HR12‐1	51 531 582	45 269 773	12.15	46.00	66.66	96.00	16.83	79.17
HR12‐2	53 342 506	48 236 985	9.57	46.00	71.97	90.80	14.90	75.90
HR12‐3	39 824 998	35 775 352	10.17	48.00	68.85	95.50	11.31	84.19
HR24‐1	48 422 048	42 815 229	11.58	45.00	69.54	95.60	17.36	78.24
HR24‐2	43 338 340	39 663 374	8.48	44.00	61.56	93.40	16.29	77.11
HR24‐3	42 433 604	38 835 749	8.48	49.00	71.40	94.90	9.51	85.39
HR48‐1	43 653 456	39 762 828	8.91	44.00	56.76	91.60	14.99	76.61
HR48‐2	37 894 414	34 315 407	9.44	44.00	55.61	92.60	15.40	77.20
HR48‐3	42 486 436	38 707 521	8.89	44.00	60.18	95.20	16.30	78.90
ZS0‐1	46 625 602	41 020 778	12.02	46.00	71.09	94.30	16.25	78.05
ZS0‐2	48 120 716	42 194 592	12.32	47.00	69.59	94.80	14.40	80.40
ZS0‐3	42 576 320	38 432 417	9.73	47.00	64.92	94.80	12.16	82.64
ZS12‐1	45 354 078	39 798 395	12.25	46.00	65.65	93.00	15.01	77.99
ZS12‐2	44 975 674	39 552 394	12.06	47.00	74.07	91.50	15.01	76.49
ZS24‐1	53 669 230	47 363 672	11.75	46.00	67.07	92.70	16.06	76.64
ZS24‐2	55 131 416	49 939 119	9.42	44.00	63.38	93.30	16.11	77.19
ZS24‐3	41 023 534	36 764 413	10.38	47.00	67.66	95.80	11.29	84.51
ZS48‐1	44 327 802	40 192 320	9.33	44.00	65.26	90.90	14.23	76.67
ZS48‐2	45 137 982	40 979 051	9.21	44.00	63.40	91.20	14.50	76.70

### Principal component analysis reveals distinct responses in HR and ZS after whitefly infestation

**Figure 2:**
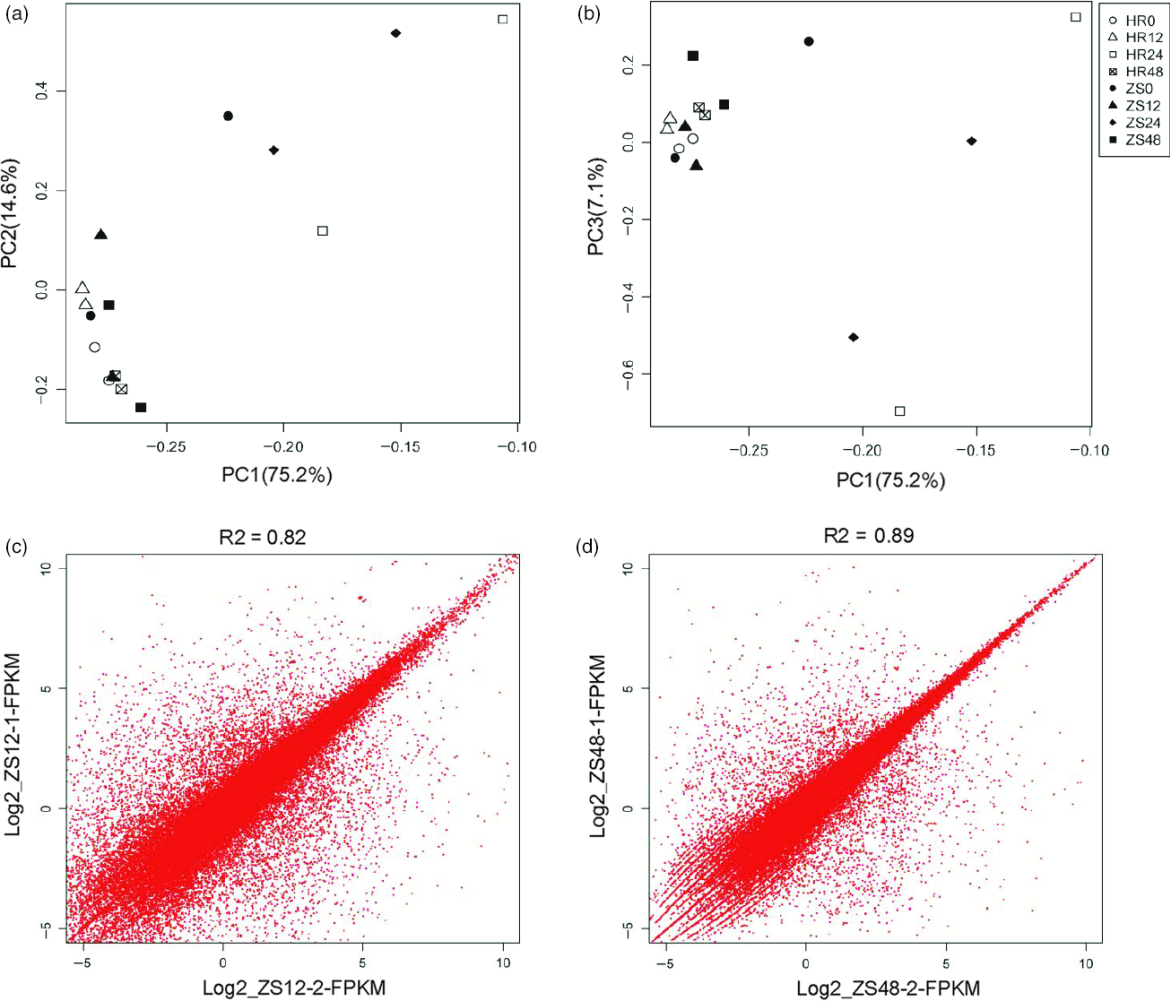
Evaluation of RNA‐Seq data quality. Principle component analysis (PCA) factorial maps showing the largest components of variance. PCA was performed using the R function “prcomp” based on normalized read counts obtained from DESeq. (a) PC1 (75.2% of the variance) and PC2 (14.6% of the variance). (b) PC1 (75.2% of the variance) and PC3 (7.10% of the variance). In (a) and (b), hours of whitefly infestation are indicated by the number following the HR or ZS cultivar such that HR0 indicates 0 h of whitefly infestation in the HR cultivar. Additional figure legend info: HR0 is depicted by empty circles, HR12 by empty triangles, HR24 by empty squares, HR48 by checked squares, ZS0 by filled circles, ZS12 by filled triangles, ZS24 by diamonds, and ZS48 by filled squares. (c) Pearson's Correlation coefficient testing the ZS12 dataset. (d) Pearson's Correlation coefficient testing the ZS48 dataset. Data are from the HR and ZS cultivars following whitefly infestation for 0, 12, 24 and 48 h.

To provide an overview of the transcriptomic landscape and reduce the dimensions of the large datasets, a principal component analysis (PCA) was performed with normalized read counts obtained from DESeq based on the prcomp function in the R environment (Figure 2a,b). Replicates of the HR12 and HR48 treatments were closely clustered on the two‐first PCs and PC3 showed all samples are concentrated. PC3 analysis revealed that a distinct and more cohesive group was formed among the HR24 and ZS24 samples as compared to the other samples (Figure S2). However, PC3 explained a relatively small proportion of the overall variance found in the data set (<9%). Differential expression analysis among the different treatments were confirmed based on PCA. In addition, the FPKM of ZS12 and ZS48 were evaluated with Pearson's Correlation Tests, which generated correlation coefficients of 0.82 and 0.89 respectively (Figure 2c,d). We also performed PCA based on per kilobase of exon model per millions mapped reads (FPKM) of all transcripts from Cufflinks. Overall, these results indicated relatively higher correlation among the different replicates and that the HR and ZS cultivars showed distinct time‐dependent responses after whitefly infestation.

### Global transcriptome changes in cotton during whitefly infestation

**Figure 3:**
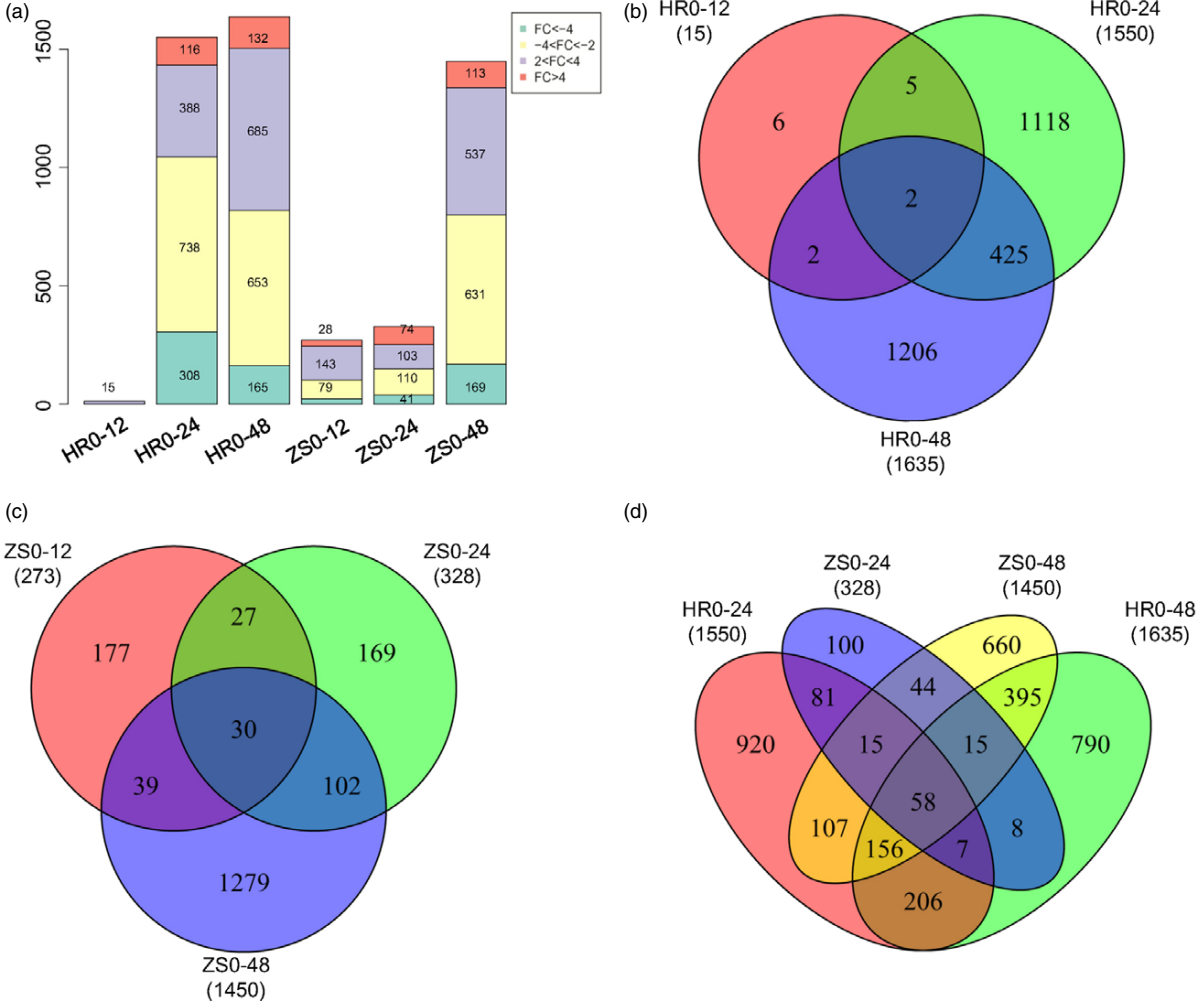
Expression dynamics change and comparative analysis of DEGs between the HR and ZS cultivars following whitefly infestation at different time points. (*P *<* *0.01 and |log2(fold‐change)| > 2). (a) Number of transcripts exhibiting changes in expression in the HR and ZS cultivars following whitefly infestation. Fold change (FC) in expression calculated as log2 of the ratio of gene expression in cotton with (i.e. at 12, 24, or 48 h) and without whitefly infestation (i.e. 0 h). The values indicated in the boxes represent the number of DEGs at different time points in the HR and ZS cultivars. Venn diagram showing common or uniquely regulated genes at different time points in the whitefly‐infested HR cultivar (b) or ZS cultivar (c). (d) Cross‐comparison Venn diagram showing the number of constitutively expressed genes following whitefly infestation in the HR and ZS cultivars.

The total mapped reads of all genes were used for differential expression analysis using DESeq [*P*‐value (*P*) < 0.01] with gene expression levels calculated as FPKM. After integrating the sequencing data from the post‐infestation libraries, a total of 3720 differentially expressed genes (DEGs) were detected in the HR and ZS cultivars at least one time point (Table S1). Scatter‐plot showed the number of DEGs between HR and ZS cultivars at different time points (Figure S3, Table S2). When compared to control (0 h), there were marginally more down‐regulated genes than up‐regulated genes in both the HR and ZS cultivars following whitefly infestation for >12 h. The number of genes exhibiting either up‐regulation or down‐regulation in the respective sample sets compared to the control HR0 and ZS0 samples are depicted in Figure 3 and consisted of: 101 down, 171 up (ZS12); 151 down, 177 up (ZS24); 800 down, 640 up (ZS48); 15 down, 1 up (HR12); 1046 down, 504 up (HR24); and 818 down, 817 up (HR48; Figure 3a).

Comparative analysis of DEGs among the three post‐whitefly infestation time points in the HR cultivar revealed limited commonality of DEGs (only two sequences) across all three time points (i.e. HR12, HR24, and HR48); much greater overlap (427 sequences) though was seen in the HR24 and HR48 time points (Figure 3b). Similar comparison using the ZS cultivar revealed generally greater overlap of the three time points with the exception of ZS24 and ZS48, which had 132 common genes (Figure 3c). In addition, DEGs were also analysed between the HR and ZS cultivars at 24 and 48 h post‐infestation (Figure 3d). We found that the greatest number of DEGs were distinctive to specific datasets (i.e. 920 in HR24, 790 in HR48, 100 in ZS24, and 660 in ZS48) with relatively little overlap (58 DEGs) across the four samples. These results suggest that gene expression between HR and ZS is dissimilar with specific genes up‐regulated/down‐regulated depending on the infestation period.

**Figure 4:**
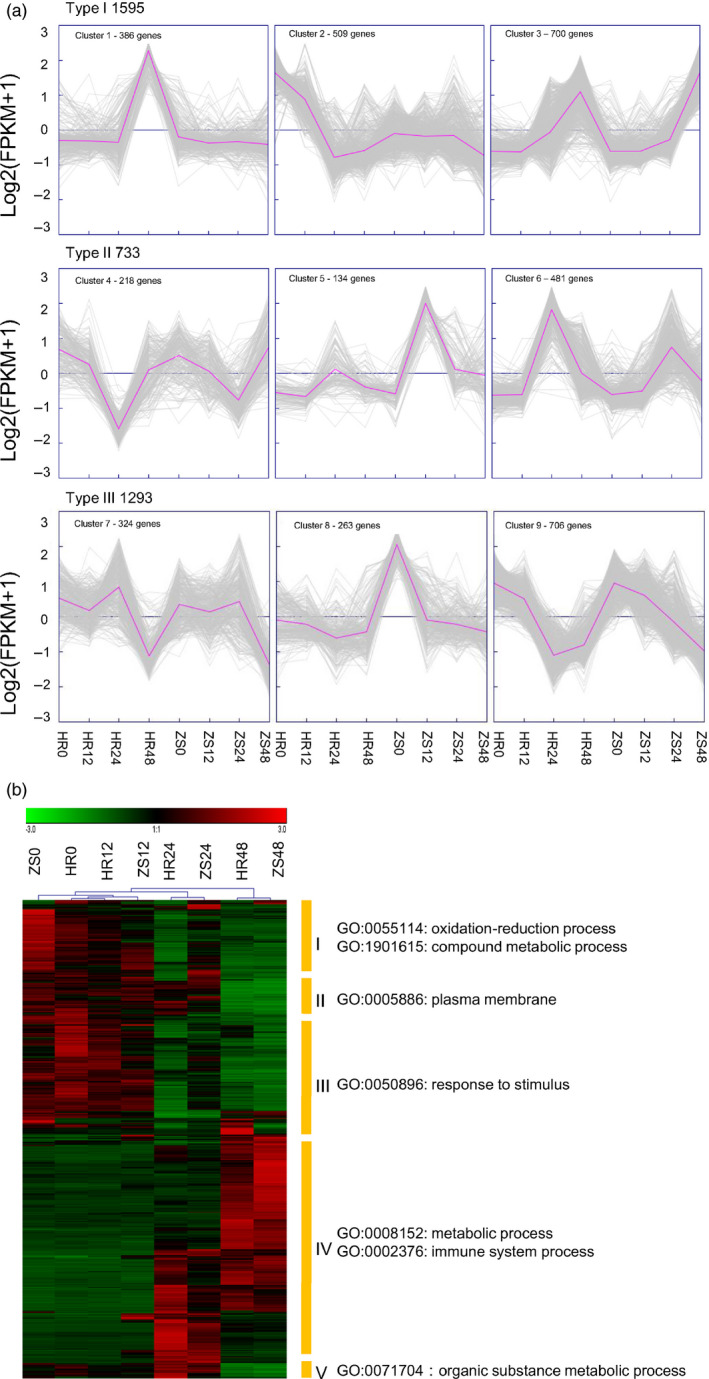
Cluster analysis of 3720 DEGs between the HR and ZS cultivars at three time points based on the K‐means method or hierarchical clustering. (a) HR0 and ZS0 are control non‐infested samples. HR12, HR24, HR48, ZS12, ZS24, and ZS48 refer to 12, 24 and 48 h whitefly infestation. In all panels, grey lines indicate the expression levels of individual genes and blue lines indicate a ‘consensus’ of all the DEGs within a specific subcluster. (b) Heatmap of 867 DEGs between the HR and ZS cultivars. Red and green colours indicate up‐ and down‐regulated genes, respectively, from both control and whitefly infested HR and ZS. Each cluster was analysed by GO enrichment. The heatmap was generated from the FPKM data.

Using Genesis software (Sturn *et al*., 2002), the expression patterns of 3720 DEGs were divided into three groups based on K‐means clustering (Figure 4a). In type I, most expression profile of 1595 DEGs were relatively stable both before and after whitefly infestation in the ZS cultivar except at 48 h, but varied significantly in the HR cultivar between 24 and 48 h post‐infestation. Type I genes were then divided into three sub‐clusters. In Cluster 1 (386 genes), most genes were stable at the all the time points in the ZS cultivar, but were stable at 0–24 h, and then up‐regulated at 24–48 h in the HR cultivar. Cluster 2 (509 genes) genes were down‐regulated at 24–48 h in the ZS cultivar, similar to the Cluster 1 genes in the HR cultivar, varied dramatically during the initial stage of whitefly infestation. Most of the cluster 3 (700 genes) genes were continuously up‐regulated in the HR and ZS cultivars at all four time‐points. Type II gene (733 DEGs) profiles were similar in both the HR and ZS cultivars. These genes were divided into three sub‐clusters. In Cluster 4 (218 genes), the gene expression profiles were similar in both the HR and ZS samples at all time points, albeit with changes in expression of this gene set more rapid in HS than ZS. In contrast, the expression profiles of the Cluster 5 (134 genes) and Cluster 6 (481 genes) genes were largely inverted between the HR and ZS cultivars within the first 24 h of infestation, but were consistently down‐regulated at 48 h post‐infestation in both cultivars. Type III genes (1293 DEGs) did not exhibit any significant differences between the two cultivars. With three sub‐clusters in type III, the expression profile of the Cluster 7 (324 genes) set was similar in both the HR and ZS cultivars. Most genes in Cluster 8 (263 genes) were stable throughout whitefly infestation in the HR cultivar, whereas they were down‐regulated in ZS. In Cluster 9 (706 genes), the gene expression profiles indicated down‐regulation at 0–24 h in the HR cultivar followed by subsequent up‐regulation at 24–48 h. However, in the ZS cultivars, these genes were down‐regulated throughout whitefly infestation.

To more clearly differentiate DEGs linked with whitefly infestation, we performed hierarchical clustering analysis on the expression pattern of 867 common DEGs that were detected after whitefly infestation and that exhibited differences in gene abundance between the HR and ZS cultivars. An False Discovery Rate (FDR) < 0.05 and an expression value of log2(FPKM) were used as standards to evaluate significant expression differences of GO enrichment between the HR and ZS cultivars throughout the whitefly infestation period (Figure 4b). Based on the hierarchical clustering analysis, the expression patterns of DEGs were clustered into five classes. Class I genes, which function in oxidation‐reduction and compound metabolic processes (i.e. beta‐galactosidase 5, tubulin beta 8, expansin protein, zinc finger proteins, heat shock proteins and cytochrome P450 superfamily proteins), were highly abundant genes that were significantly down‐regulated in the HR and ZS cultivars at 48 h post‐infestation. The expression profile of these DEGs, however, was inverted in the HR and ZS cultivars at 24 h post‐infestation. The 75 DEGs comprising the class II genes (i.e. pentatricopeptide repeat‐containing protein, *LAC14* and SOUL heme‐binding family proteins) showed similar expression patterns in both cultivars throughout the whitefly infestation period. Class III (i.e. *ERF8*,* TPS21*) genes began to exhibit down‐regulation in both the HR and ZS cultivars at 24 h post‐infestation and were significantly down‐regulated at 48 h. Class IV genes (i.e. plasma membrane intrinsic protein, oxidative stress 3 and pathogenesis‐related protein) were largely up‐regulated in both cultivars at 48 h post‐infestation; however, a subset of genes in this GO class were specifically up‐regulated within the 24 h time period (Table S3). Overall, the expression profile of DEGs present in both cultivars was consistent with up‐regulation or down‐regulation apparent in HR and ZS at the same period of infestation. However, the expression profile of numerous genes unique to the respective HR and ZS cultivars exhibited inverse expression profiles. These differences in expression may account for the contrasting resistance phenotypes in the two cultivars.

### Functional annotation of DEGs and pathway enrichment analysis

**Figure 5:**
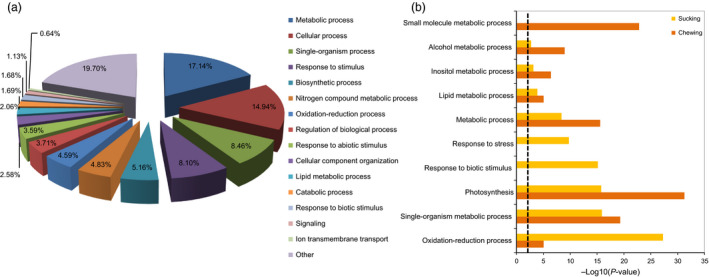
Analysis of GO enrichments. (a) Functional classification of all DEGs following whitefly infestation in the HR and ZS cultivars. Pie chart represents differences in GO term enrichment. (b) Common and unique GO enrichments for various biosynthetic processes detected in cotton infested with phloem‐sucking or chewing insects (*P *<* *0.01).

The significant DEGs were further annotated based on functional gene ontology using default Blast2GO parameters (Conesa *et al*., 2005). The transcriptomic data obtained in this experiment were compared to the TAIR 10 and all KO protein databases using BLASTx at an e‐value of 10^−5^ for KEGG pathway annotations. GO assignments were used to classify the functions of the DEGs by Blast2GO (Fisher's exact test, FDR < 0.05). Based on multiple testing corrections, 3720 DEGs described previously were categorized into 123 functional groups (Table S4). The 3270 DEGs comprise three major enrichment categories: 61 biological processes (BP), 14 cellular components (CC), and 45 molecular functions (MF). Genes associated with metabolic process (17.4%), nitrogen compound metabolic process (4.8%), and lipid metabolic process (2.1%) were all grouped into metabolic processes, which were the most abundant functional gene group (Figure 5a). Response to stimulus (8.1%), oxidation‐reduction processes (4.6%), response to abiotic stimulus (3.59%), and response to biotic (1.7%) were grouped under the plant host defense category, which involves plant responses to various abiotic and pathogen/insect stresses. In addition to these two major categories, additional categories identified corresponded to biosynthetic process (5.2%), signalling (1.2%), transmembrane transport (0.7%) and two cell structure groups that include cellular process (14.9%) and cellular components (2.6%). Publically available transcriptomic data (Bioproject ID: PRJNA245406) for cotton infested for 48 h with chewing insects such as the cotton boll weevil (*Anthonomus grandis*) were also analysed. GO enrichment data for cotton host responses to the two different pest‐feeding modes (whitefly sap‐sucking *vs*. lepidopteran chewing) were compared. These analyses indicated that 19 GO biological process enrichments were common. The ten most abundant GO enrichments in cotton plants infested by chewing or sap‐sucking pests are summarized in Figure 5b. Genes related to oxidation‐reduction, response to stress and response to biotic stimulus had significantly higher significance levels in plants infested with sap‐sucking pests than those with a chewing pest (*P *<* *0.001). In contrast, photosynthetic, small molecule metabolic and alcohol metabolic process were more enriched in cotton infested with chewing pests. Interestingly, responses to biotic stimulus processes in cotton were significantly higher in cotton infested with the sap‐sucking pest (Figure 5b). In the HR cultivar, stress process, DNA metabolic process, response to oxidative stress, cellular component biogenesis and cell wall modification predominated following whitefly infestation. While, in the ZS cultivar, the major biological process sub‐categories were: organic hydroxy compound metabolic process, nucleotide biosynthetic process, ion transmembrane transport, ATP biosynthetic, cellular nitrogen compound biosynthetic and other cellular processes (*P *<* *0.001; Table 2). In the susceptible cultivar, transcription factors such as *ERF1*, WRKYs and MYBs were most abundant during the initial whitefly infestation stage (Figure S5).

**Table 2:** GO enrichment (biological processes, FDR < 0.05, Blast2GO, Fisher's exact test) were analysed in the HR and ZS cultivars following whitefly infestation for 12, 24, and 48 h. ‘‐’ indicates the absence of any enriched GO terms

GO‐ID	Term	HR		ZS	
		DEG	−log (*P*‐value)	DEG	−log (*P*‐value)
Stress response					
GO:00010607	Response to biotic stimulus	40	12.72	35	8.27
GO:0009628	Response to abiotic stimulus	24	2.55	22	1.49
GO:00061052	Defense response	70	4.08	–	–
GO:00061050	Response to stress	173	7.55	–	–
GO:0055114	Oxidation–reduction process	441	24.07	412	11.57
GO:000610710	Response to oxidative stress	45	4.08	–	–
Metabolism synthesis					
GO:0044710	Single–organism metabolic process	660	13.34	687	10.66
GO:0006259	DNA metabolic process	83	2.34	–	–
GO:0006020	Inositol metabolic process	7	1.68	9	3.00
GO:0006629	Lipid metabolic process	139	2.33	150	2.60
GO:0046034	ATP metabolic process	–	–	40	9.24
GO:1901615	Organic hydroxy compound metabolic process	–	–	16	2.24
Nucleinic acid and protein					
GO:0006334	Nucleosome assembly	40	6.30	–	–
GO:0040031	snRNA modification	4	1.68	4	1.60
GO:0071824	Protein–dna complex subunit organization	40	6.00	–	–
GO:0070271	Protein complex biogenesis	56	2.52	–	–
GO:0009165	Nucleotide biosynthetic process	–	–	46	4.55
Cell					
GO:0042545	Cell wall modification	49	4.34	–	–
GO:0034622	Cellular macromolecular complex assembly	59	3.82	–	–
GO:0044085	Cellular component biogenesis	74	1.92	–	–
GO:0044271	Cellular nitrogen compound biosynthetic	–	–	389	1.32
Other					
GO:001510710	Photosynthesis	74	13.28	243	5.47
GO:0006754	ATP biosynthetic process	–	–	38	10.09
GO:0034220	Ion transmembrane transport	–	–	67	4.82
GO:0015992	Proton transport	–	–	49	5.74
GO:0006811	Ion transport	–	–	154	3.05

**Figure 6:**
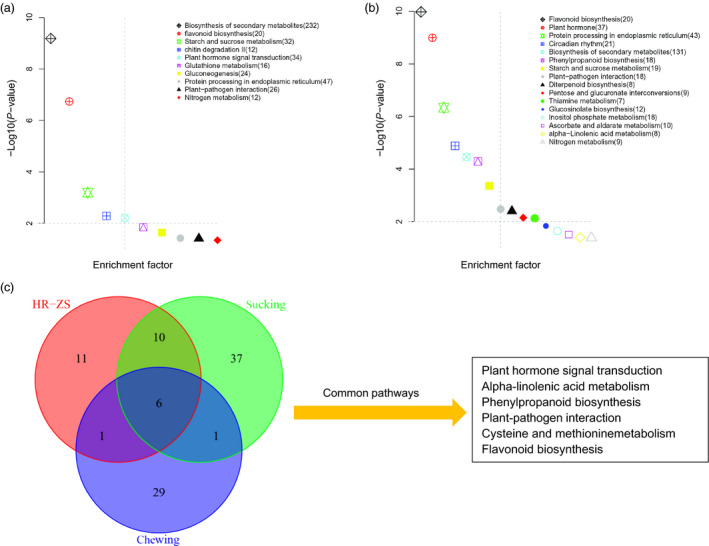
Distribution of KEGG pathways. Data were visualized using a scatter diagram of KOBAS2.0 results with *P*‐value levels indicated by “−log10 (*P*‐value)” and an enrichment factor indicative of individual pathways. Parentheses values in the figure legend represent the number of each pathway's components present in the DEG dataset. (a) Total number of DEG pathways in the HR cultivar. (b) Total number of DEG pathways in the ZS cultivar. (c) Ven diagram showing the overlap of common and unique pathways present in publically available transcriptomic datasets and transcriptome data from this study. The “chewing” dataset is for cotton flower buds infested by boll weevil larvae, whereas the “sucking” dataset is for cotton leaves infested with aphids and whiteflies. The most highly represented pathways (*P *<* *0.01) are indicated to the right of the diagram.

For further functional categorization, KEGG pathway analyses were performed using the KOBAS2.0 database such that all of the DEGs from both the HR and ZS cultivars after whitefly infestation were assigned to 28 KEGG pathways (*P *<* *0.05). In the HR cultivar, DEGs were assigned to 11 significant KEGG pathways, whereas DEGs were assigned to 23 significant KEGG pathways in the ZS cultivar (Table S5). Several pathways were associated with plant responses to herbivore infestation; some of the pathways described in this report are consistent with those identified in other reports. For example, variations in plant hormones and metabolic synthesis transcripts following infestation by aphids and whiteflies have been reported previously (Cho *et al*., 2005; Jin *et al*., 2011; Moran and Thompson, 2001). Major pathways related to plant responses following whitefly infestation of HR include: biosynthesis of secondary metabolites (232), flavonoid biosynthesis (20), starch and sucrose metabolism (32), chitin degradation (12) pathways (*P *<* *0.01; Figure 6a). Pathways unique to the whitefly‐infested ZS cultivar include phenylpropanoid biosynthesis (18), linoleic acid metabolism (8) and ascorbate/aldarate metabolism (10). Pathways common (*P *<* *0.05) to both HR and ZS include secondary metabolites, plant hormone, flavonoid biosynthesis, plant‐pathogen interaction, nitrogen metabolism, starch and sucrose metabolism, and protein processing in endoplasmic reticulum (Figure 6b, Table S5). Based on the GO function and KEGG pathway analyses, we identified multiple genes related to cell wall modification and metabolite assimilation that exhibited dramatic variation in transcript levels at the initial stage of whitefly infestation. In contrast, transcripts associated with secondary metabolites, transcription factors and plant hormone‐related genes exhibited significant changes in gene expression levels at the later time points in infestation. We also used KOBAS2.0 to analyse two publically available DEG datasets (generated from Roche GS‐Titanium 454 sequence data) for whitefly and aphid (both sap‐sucking insects). Based on these data, transcripts involved in signal transduction, phenylpropanoid biosynthesis and plant**–**pathogen interactions appear to be among the most important pathways in plant responses to pest infestation, of those pathways, 11 were unique to the whitefly infested HR and ZS cultivars (Figure 6c).

### Data integration and network construction

**Figure 7:**
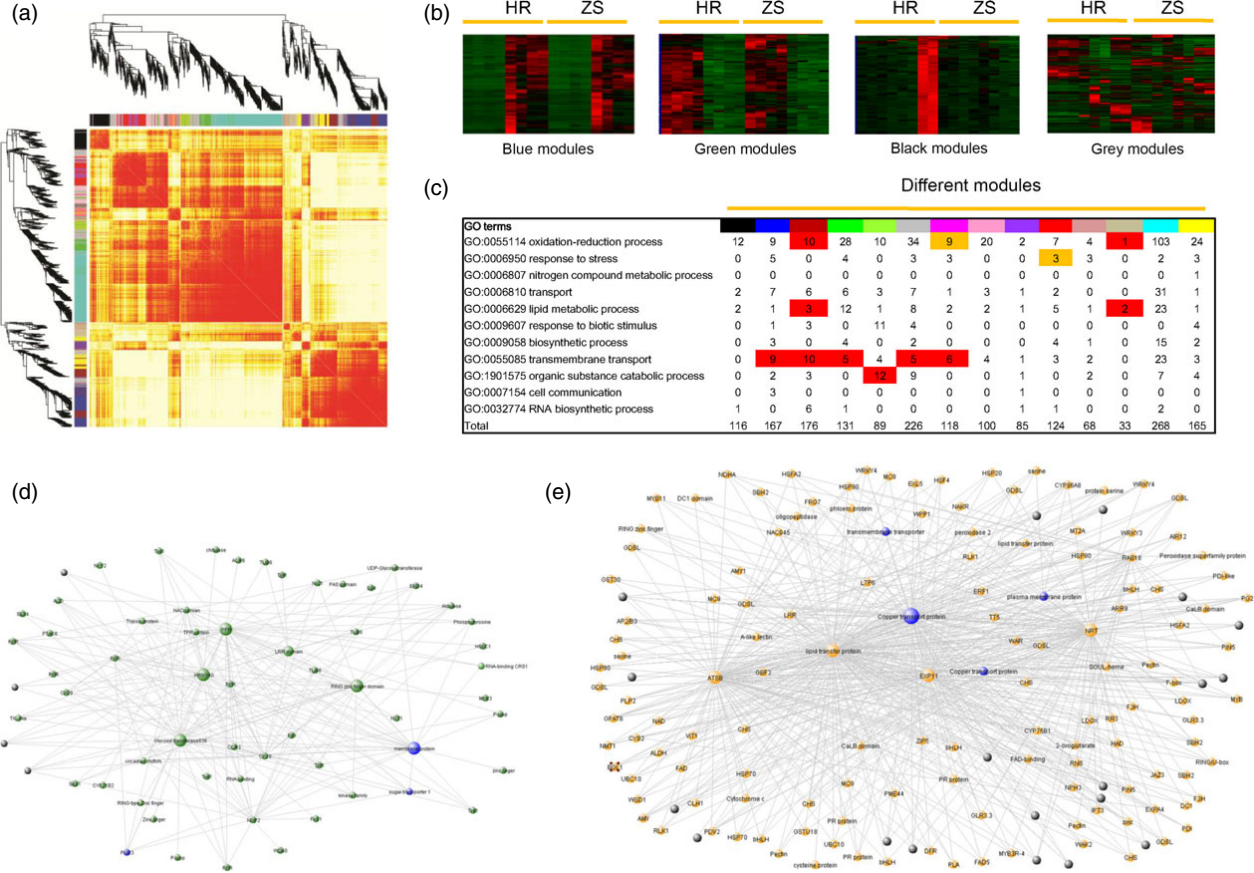
Weighted gene co‐expression network. (a) Heatmap plot of the gene network. Light colours represent low overlap and progressively darker red colours represent higher overlap of gene pairs from the respective datasets. Different modules are indicated by the diagonals. (b) Example of four modules identified in the heatmap and functional annotation enrichment of DEGs in the respective network modules. For each module, the number of DEGs clustering with each related biological process is shown. Processes with statistically significant enrichment values based on Fisher's exact test are highlighted in red (*P *<* *0.01) and green (*P *<* *0.05). (c) Fourteen modules were GO enrichment analysis by Fisher's exact test. (d) Most highly connected gene networks in the module of up‐regulated DEGs after whitefly infestation. Blue nodes represent GO enrichment terms, whereas black nodes represent genes of unknown function (e) Down‐regulated DEGs network in green module.

To determine potential relationships between modules (co‐expression genes) and traits, which is useful for identifying activation of specific biological processes, we constructed a weighted gene co‐expression network based on log2 (FPKM) values for 3720 DEGs identified in our RNA‐Seq data. A weighted gene network was obtained by using a soft thresholding power (β = 8; cut‐off = 0.85) with higher adjacency (Figure S6). A heatmap‐based plot incorporating different module assignments and gene dendrograms was used to initially visualize the Topological Overlap Matrix (TOM) of DEGs following whitefly infestation (Figure 7a). Based on this figure, it became clear that each module had higher overlaps so the network was divided into 14 modules. Hierarchical clustering, GO enrichment and assignment of gene number revealed different characteristics for the modules (Figures 7b,c and S6). Genes in the blue module were up‐regulated after whitefly infestation and are highly correlated with *WRKY40*, glycosyl transferases and zinc finger domain proteins, which appear to hub genes that play a role in insect/host plant interactions (Figure 7d). The green module revealed genes down‐regulated following whitefly infestation, including five hub genes and a number of transcripts potentially involved in transmembrane transport (Figure 7e). Plant**–**pathogen interactions trigger regulation of primary plant metabolism genes (Rojas *et al*., 2014). Our network constructions showed high correlation with similar gene sets, notably lipid‐transfer proteins and nitrate transmembrane transporters as well as genes potentially involved in oxidation‐reduction, lipid metabolism and organic substance catabolism (Figure 7c).

### Genes potentially involved in cotton resistance to whitefly infestation

**Figure 8:**
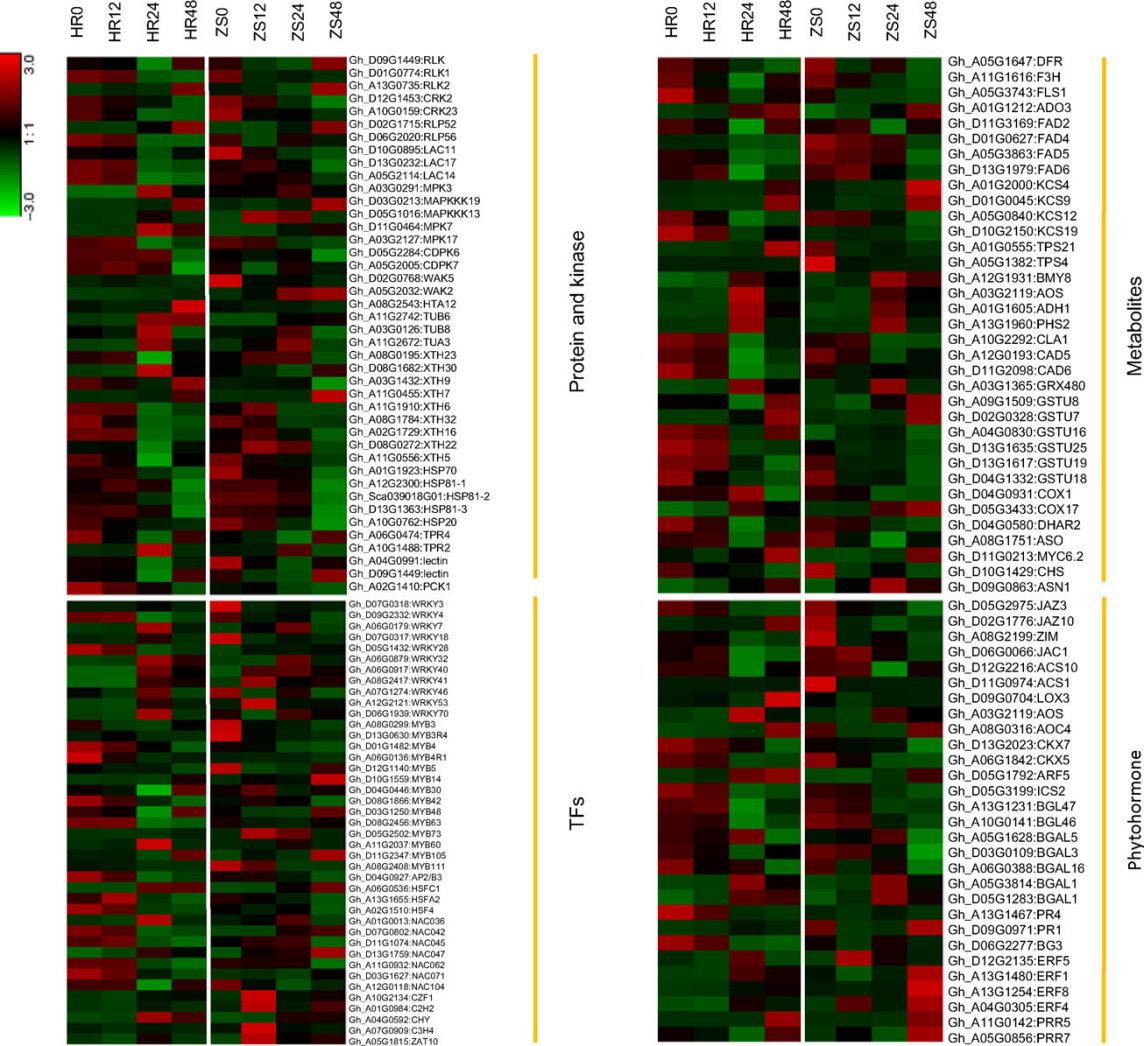
Heatmap of clustered GO terms for genes potentially linked to resistance that are differentially expressed in whitefly infested HR and ZS cultivars.

Our combined KEGG pathway and GO enrichment analyses identified a diverse group of defense‐related genes that undergo differential transcriptional responses following whitefly infestation. A majority of these genes belong to the Type II and Type III groups described in Figure 4 (see also Table S1), which include protein kinases, transcription factors, metabolite synthases, and hormone and pathogen‐related genes (Figure 8, Table S6). Gene families of particular interest as potential targets for engineering pest resistance are further elaborated below.

### Protein kinases and protein

The roles of kinases are well‐documented in pathogen and insect‐induced plant immunity. Among the most critical kinases are the receptor‐like kinases (RLKs) that trigger the initial plant immune responses (Heese *et al*., 2007). A search of our DEGs data revealed seven cysteine‐rich RLKs, some of which (*RLK1* and *CRK2/23*) were down‐regulated during the later stages of whitefly infestation in both the HR and ZS cultivars. In general though, RLK family genes were significantly up‐regulated in HR, but down‐regulated in ZS during the initial stage of whitefly infestation. In contrast, a number of protein kinases (*MPK3/7*,* MAPKKK13/19* and *CDPK*s) were up‐regulated, whereas cell wall‐associated kinases (*WAK2*,* WAK5*) were down‐regulated by whitefly infestation, as were cell wall‐modifying enzymes such as xyloglucan endotransglucosylase/hydrolase proteins (*XTH9*,* XTH22*,* XTH32*). The genes described above are involved in several pathways such as protein processing in endoplasmic reticulum, plant–pathogen interaction and plant hormone signal transduction during biotic stress (Figure 8).

### Transcription factors

Transcription factors are important regulators of both biotic and abiotic stress responses by turning on or off the plant immune defense system (Nakashima *et al*., 2009). Multiple transcription factors were identified among the DEG data including WRKY, zinc finger, MYB, ERF/AP2, the bZIP/HD‐ZIPWD‐40 repeat family proteins, NAC domain, bHLH and GRAS. Some of these transcription factors have previously been reported to be critical components of the plant adaptive response to biotic stress. The expression of a majority of the transcription factors, including NAC domain proteins and most MYBs, was suppressed after whitefly infestation. Of the 30 WRKYs identified, *WRKY40*,* WRKY41* and *WRKY53* were significantly up‐regulated in ZS during the initial infestation period, whereas WRKY3, *WRKY46* and *WRKY70* were down‐regulated. Other zinc finger‐containing transcription factors from the C2H2, C3H and CCT domain protein family were also up‐regulated during the later stage of infestation. Among the 25 MYBs were identified, the expression profiles of *MYB111*,* MYB73* and an *AP2/B3* type were similar at all time points in HR and ZS. In addition, *ERF1* and heat‐shock transcription factor (HSF), which are crucial in phytohormone signalling, were also detected in our transcriptome data. Detailed information on the expression profiles of these transcription factors is summarized in Figure 8.

### Metabolism and oxidative stress

Metabolite synthesis‐related genes may play roles in herbivore defenses. We identified a subset of genes participating in multiple branches of the phenylpropanoid pathway, including biosynthesis of flavonoids, phenolic compounds and the terpenoid backbone. Expression of many of these genes was down‐regulated in the HR cultivar 24 h after infestation, but remained stable in the ZS cultivar.

Reactive oxygen species (ROS) play an important role in plant responses to biotic stresses such as insect infestation (Liu *et al*., 2010). Glutathione is a major ROS scavenger in plants and is important in biotic stress tolerance (Gill and Tuteja, 2010). The expression of enzymes involved in glutathione synthesis, namely, cytochrome oxidase (*COX1*,* COX17*), glutathione S‐transferase (*GSTU*), and plant L‐ascorbate oxidase (*ASO*) varied dramatically during whitefly infestation. For example, *COX1* was highly down‐regulated at all time points and *COX17* was highly up‐down‐regulated at late stages (24–48 h) in ZS. The expression of other ROS‐related genes, such as plant invertase, thioredoxin superfamily protein *GRX480* and cinnamyl alcohol dehydrogenase (*CAD*), however, was not as evident as their transcript levels fluctuated during whitefly infestation. Taken together, the results suggest that expression of genes related to the phenylpropanoid and flavonoid pathways are extremely dynamic during whitefly infestation (Figure 8).

### Phytohormone signalling related genes

Genes involved in different phytohormone signalling pathways were also characterized. Several signalling pathways, including ethylene signal transduction‐related genes encoding ACC synthase (*ACS1*) and *ERF1* showed distinct expression patterns in the HR and ZS cultivars. Five jasmonate zim‐domain genes (*JAZ3*,* JAZ6*,* JAZ10* and *JAC1*) that function as repressors in JA signalling were more highly suppressed in HR24 than in any of the other samples. In addition, jasmonate signal‐related genes, jasmonic acid carboxyl methyltransferase, allene oxide synthase (AOS), and allene oxide cyclase (*AOC4*) were highly expressed after 48 h infestation, while lipoxygenases (*LOX3*) was down‐regulated. Genes associated with defense responses mediated by SA, a well‐known phytohormone involved in the induction of pathogen resistance, were down‐regulated in the ZS cultivar. In addition, two genes encoding pathogenesis‐related proteins (*PR1*,* PR4*) were significantly suppressed after whitefly infestation in HR24. In both HR and ZS, the expression of two cytokinin oxidases (*CKX5*,* CKX7*), which are part of the cytokinin signalling pathway, were suppressed at the 48 h time point. Abscisic acid (ABA) and auxin signalling pathway genes, such as *ARF5*, were up‐regulated in both HR and ZS at the later stages of whitefly infestation.

### Quantitative real‐time PCR validation of RNA‐Seq data

**Figure 9:**
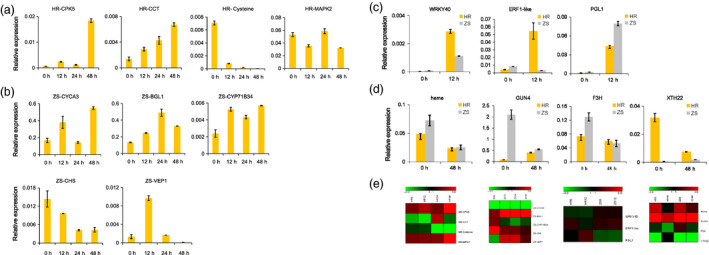
qRT‐PCR validation of selected DEGs in the HR and ZS cultivars following whitefly infestation. (a) Expression level of four DEGs in the HR cultivar in response to whitefly infestation over time (12, 24, and 48 h). The 0 h time point (i.e. no whitefly infestation) serves as a control. (b) Expression level of five DEGs over time following whitefly infestation in the ZS cultivar. (c) Expression level of three DEGs in HR and ZS after12 h whitefly infestation. (d) Expression level of four DEGs in HR and ZS following 48 h whitefly infestation. Relative expression was calculated based on the 2^−ΔCt^ ratio using *GhUBQ7* as the reference gene. Three biological replicates and three technical replicates were performed. Error bars represent standard error of genotype means. (e) These genes corresponding to expression patterns in RNA‐Seq data.

To confirm expression of the DEGs, we used qRT‐PCR to examine the transcriptional profile of 16 genes (Table S7). Validation of these genes was performed using three independent biological replicates. In the HR cultivar, *CPK5*, which was up‐regulated at all time points after infestation in the DEG analysis, exhibited a significant increase (29‐fold) in expression 48 h after whitefly infestation. In contrast, the expression of *CCT* increased steadily over the duration of the infestation period (i.e. 12–48 h), whereas cysteine protease superfamily protein was down‐regulated, and *MPK2* showed a slight change compared to the respective controls (Figure 9a). Similarly, in the ZS cultivar, cyclin‐dependent protein kinase 3 (*CYCA3*), β‐glucosidase (*BGL1*), cytochrome P450‐71B34, chalcone and stilbene synthase (*CHS*), and NAD(P)‐binding protein (*VEP1*) exhibited dramatic variations in expression when compared to whitefly‐free controls (Figure 9b).

We next directly compared the expression profile of select DEGs in the HR and ZS cultivars at specific points in whitefly infestation. Three genes were selected for analysis in the 12 h samples. The gene levels of all three genes were higher following infestation in both cultivars (Figure 9c). *WRKY40* and ERF‐like expression were elevated in the resistant HR cultivar compared to the susceptible ZS, whereas *PGL1* expression was higher in ZS (Figure 9c). Four genes were selected for analysis in the 48 h samples. In the HR cultivar, both heme‐binding protein and *F3H* were highly expressed prior to infestation and underwent only moderate decreases in expression following infestation (Figure 9d). In ZS, however, *F3H* expression was dramatically reduced following whitefly infestation. The expression profiles of *GUN4* and *XTH22* exhibited significant variation and were inverted in the two cultivars. *GUN4* expression, which was significantly higher in ZS than HR prior to whitefly introduction, decreased after 48 h whitefly infestation in ZS but increased in the HR cultivar (Figure 9d). *XTH22* expression in contrast was the exact opposite, with higher levels in HR prior to whiteflies followed by a significant reduction after whitefly infestation, whereas transcript levels in the ZS cultivar increased in response to the whiteflies (Figure 9d). These genes expression patterns were showed from RNA‐seq results in Figure 9e. Overall, the expression profile of most of the genes selected for qRT‐PCR validation was consistent with the DEG data, suggesting that the RNA‐Seq data are reliable and reproducible (Figure S7).

### VIGS‐mediated silencing of *GhMPK3* enhances cotton susceptibility whitefly infestation

**Figure 10:**
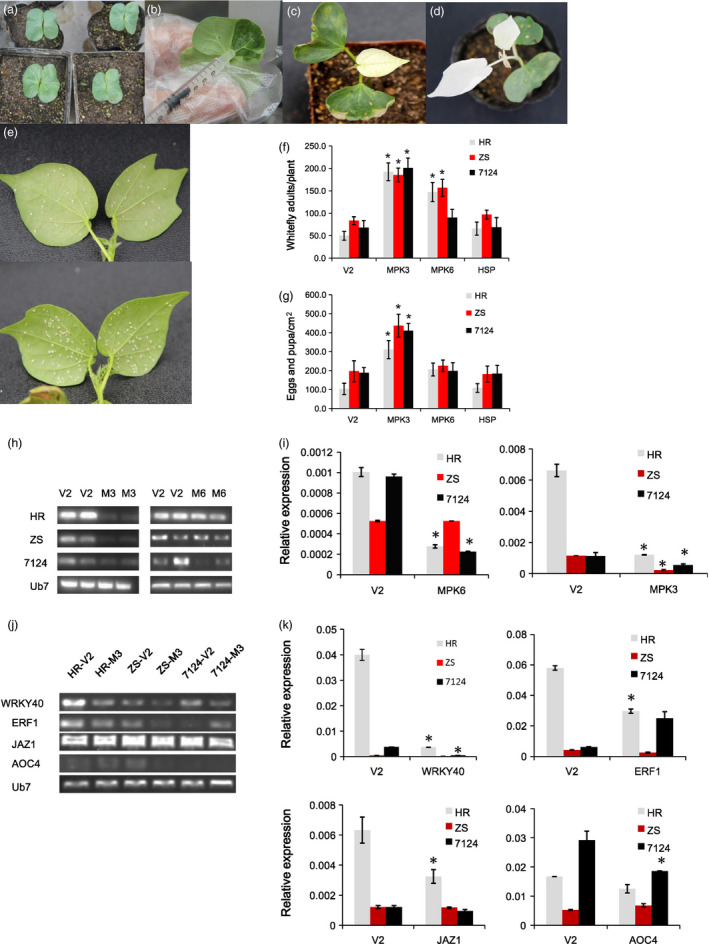
Silencing *GhMPK3* in cotton by VIGS results in enhanced susceptibility to whitefly. (a) 10‐day old young plantlets used for VIGS. (b) Cotyledons (abaxially) were used for agro‐infiltration by a syringe without a needle. (c) *TRV:GhCLA1* cotton plants exhibiting the expected leaf bleaching phenotype. The phenotype was observed 10 days after VIGS treatment thus confirming the functionality and effectiveness of the approach. (d) Bleaching phenotype of a *TRV:GhCLA1* cotton plant maintained for 1 month. (e) Representative image of the low whitefly density observed on a *TRV::00* HR plant and heavy whitefly colonization on a *TRV:GhMPK3* HR plant. (f) Quantification of whitefly adults on VIGS plants 2 weeks after infestation. (g) Quantification of whitefly eggs and pupa on VIGS plants 2 weeks after infestation. RT‐PCR (h) and qRT‐PCR (i) analysis showing down‐regulation of *GhMPK3* and *GhMPK3* in the *TRV::GhMPK3* plants. RT‐PCR (j) and qRT‐PCR (k) analysis of the expression levels of GhMPK3‐related genes in *TRV::GhMPK3* plants.

Several cotton genes that increased in expression following whitefly infestation are promising candidate genes for the observed resistance. Consequently, we selected three genes, *GhMPK3*,* GhMPK6* and *GhHSP20*, for functional analysis using VIGS with *GbCLA1* as a reporter gene to evaluate the efficiency of VIGS. Ten‐day‐old young plantlets with flat and intact cotyledons were determined to be ideal targets for agro‐infiltration (Figure 10a). At this stage, the cotyledons are tender and easily penetrated by *Agrobacterium* (Figure 10b). Most importantly, the target genes to be silenced will exhibit a clear phenotype when the true leaves emerge (Figure 10c). In these experiments, the albino phenotype of *TRV:GbCLA1* plants was maintained for more than 1 month (Figure 10d) suggesting that VIGS was efficient and durable. Ten days after VIGS treatment, *TRV:GhMPK3*,* TRV:GhMPK6* and *TRV:GhHSP20* plants from the HR, ZS cultivars were transferred to a greenhouse and infested with whiteflies for 2 weeks. While heavy whitefly colonization was observed in most of the *TRV:00* cotton plants, whitefly densities were lowest on HR (Figure 10e). Significantly higher densities (186–201 adult whiteflies per plant) were observed in the *TRV:GhMPK3* plants, which were almost three times higher than in the *TRV:00* group (50–84 adult whiteflies per plant). In the *TRV:GhMPK6* plants, there was a moderate increase in whitefly counts, whereas there was no significant change in the number of whiteflies on the *TRV:GhHSP20* plants 2 weeks after whitefly infestation (Figure 10f). In addition to adult whitefly numbers, we also examined the effects of knockdown on eggs and pupa. A significantly higher density of eggs/pupa (311–437 eggs/pupa/cm^2^ leaf) were found in *TRV:GhMPK3* plants (Figure 10g) as compared to *TRV:00* (103–196 eggs/pupa), *TRV:GhMPK6* (198–226 eggs/pupa) and *TRV:GhHSP* (109–184 eggs/pupa). Among the lines (i.e. *TRV:00*,* TRV:GhMPK6* and *TRV:GhHSP*), there were no significant differences in the densities of eggs and pupa (Figure 10g).

In parallel experiments, the expression level of two target genes, *GhMPK6* and *GhMPK3*, in VIGS‐treated plants were assessed by RT‐PCR and qRT‐PCR. RT‐PCR analysis revealed that *GhMPK3* transcripts were reduced in the *TRV:GhMPK3* plants (Figure 10h) with *GhMPK3* transcription levels an order of magnitude lower than in the *TRV:00* plants (Figure 10i). For *GhMPK6*, transcription levels varied among the different cultivars. In the HR and 7124 cultivars, *GhMPK6* was dramatically down‐regulated in VIGS plants, but there was no significant change in the ZS cultivar (Figure 10i). Overall, the qRT‐PCR data are consistent with our RT‐PCR data. Because whitefly densities in *TRV:GhMPK3* plants were significantly increased during whitefly infestation, the expression levels of *WRKY40*,* ERF1*,* JAZ1* and *AOC4* (all possible MPK target genes or MPK pathway related genes) were analysed in control and VIGS plants (Figure 10j,k). All four genes were substantially down‐regulated in HR after silencing *MPK3*, whereas no obvious changes were observed in ZS plants when compared to vector alone controls. Notably, *WRKY40* was dramatically down‐regulated in both HR, ZS and 7124 plants. These results suggest that the enhanced susceptibility to whiteflies observed in the VIGS cotton lines was most likely due to suppression of MPK‐WRKY‐JA signalling and ET pathways.

## Discussion

### Generation of a comprehensive transcriptome from whitefly infested cotton

Cotton is an economically important crop, and phloem‐feeding insects such as whiteflies cause significant economic losses. Although some cotton cultivars resistant to cotton bollworm have been generated by genetic transformation including RNAi technology (Jin *et al*., 2012, 2015), the molecular mechanisms of insect resistance in cotton are still unclear. Dubey *et al*. (2013) found that cotton exhibit similar responses to whitefly and aphid infestations and that cotton plants responded to whiteflies more rapidly than to aphids. In that report, however, a single cotton cultivar was used for insect infestation and only 3.8 million RNA‐Seq reads were generated (Dubey *et al*., 2013).

The distinct genetic background of the ZS and HR cultivars provided a solid foundation for identifying genes involved in the cotton defense response. In addition to the merits of the selection assay for testing the cotton cultivars, an experimental approach within a controlled container (Figure S1) was designed to compare the resistance mechanisms between the HR and ZS cultivars at different time points (0, 12, 24, 48 h after whitefly infestation). To our knowledge, whiteflies are a unique insect species with high mobility and tiny size, two factors that complicate their use in controlled settings. Consequently, a considerable amount of time was spent designing preliminary experiments that established a controlled and reproducible system. We observed a high correlation in the RNA‐Seq data with two of our biological replicates, suggesting that our whitefly/cotton interaction system was robust and reliable, and would provide a valuable resource for studying plant host interactions with diminutive sap‐sucking insects.

Two methods, DESeq and cuffdiff, were utilized to fully assess the RNA‐Seq differential expression data (Anders and Huber, 2010; Trapnell *et al*., 2012). DEG counts were prioritized relative to DESeq followed by two methods for assessing DEG distribution (Figure S4), which were then validated by qRT‐PCR. Differential expression analyses revealed that more DEGs were present in the HR cultivar than ZS at all the time points, and that DEGs gradually increased with infestation time in HR. More down‐regulated genes were identified than up‐regulated in most of the HR and ZS samples at 24 h. These results are supported by previous studies reporting that infestations of whiteflies and aphids drive transcriptional suppression over induction (De Vos *et al*., 2005; Dubey *et al*., 2013). However, our results contrast with that observed in cotton damaged by chewing insects, such as the cotton boll weevil (Artico *et al*., 2014), in which up‐regulation > down‐regulation. The different types of damage that chewing and sap‐sucking insects inflect may explain the discrepancy in the respective transcriptional responses by cotton. GO enrichment and KEGG pathway analysis indicated that metabolic processes, transcription factors, transmembrane transport and responses to stress related genes might be involved in host plant resistance to infestations by phloem‐feeding insects. Comparison of our data on phloem‐feeding insect infestation with publically available (i.e. NCBI) data on chewing insects suggested that expression of genes related to oxidative phosphorylation and responses to biotic stimuli were significantly higher in cotton infested by whiteflies than infested by a chewing insect. Several transcription factors such as *WRKY40* and *NAC045* were identified and shown to form complex connections with other genes (Figure 7). The results shown in Figure 7d,e indicated that *WRKY40*, copper transport protein, P450 superfamily proteins and NRT may be hub genes that regulate cotton defenses to whitefly infestation. Previous studies have suggested that the P450 superfamily is an essential part of the plant response to biotic stress (Lee *et al*., 2010; Li *et al*., 2003).

### Insect resistance pathways in cotton are similar to those used in pathogen resistance

Blast2GO analysis of the DEGs generated a representative overview of the cotton response whitefly infestation. These results indicated that biological processes related to stress responses, secondary metabolism synthesis, hormone signalling, systemic acquired resistance, protein kinases, cation transport, transmembrane transport and ROS were significant at all time points in the ZS cultivar. Several studies have revealed that ROS are implicated in both pathogen and herbivory‐induced responses in plants (Liu *et al*., 2010; Wu and Baldwin, 2010). KEGG analysis also suggested that ROS and oxidative phosphorylation pathways were significant (*P *<* *0.01; Figure 6). The transcription of multiple genes related to ROS scavenging and redox homeostasis, such as the *GSTU* family protein cinnamyl alcohol dehydrogenase (*CAD5*) were down‐regulated in response to whitefly infestation, suggesting that whitefly feeding may induce the accumulation of ROS (Kempema *et al*., 2007). RLKs are well‐known to be involved in the perception of pathogen‐derived elicitors (Liebrand *et al*., 2013; Montesano *et al*., 2003). RLKs are transmembrane proteins with putative extracellular domains and intracellular protein kinase domains that perceive external signals and initiate signal cascades (Gou *et al*., 2010). RLKs/RLPs were more abundant in the ZS cultivar compared to the HR cultivar, suggesting a potential role in conferring the resistance phenotype. Furthermore, Ca^2+^ has been recognized as an important second messenger involved in many plant signalling actions including responses to herbivory, pathogens, and stress responses (Wu and Baldwin, 2010). Ca^2+^ signalling related genes, such as calcium‐dependent protein kinases (*CDPK6*), Ca^2+^‐binding proteins (*EDA310*), calcium‐binding EF‐hand family protein and calmodulins, were elevated during the early infestation stage with significantly higher levels in ZS than in HR. These genes were dramatically up‐regulated at the initial infestation stage and then down‐regulated during the later infestation stages. It has been shown that mechanical wounding and insect damage will trigger a transient increase in cytosolic Ca^2+^ levels and that these second messengers active subsequent signalling pathways (Howe and Jander, 2008; Lecourieux *et al*., 2006).

Transcription factors are important regulators that control the expression of multiple genes in response to slight biotic and abiotic stresses (Singh *et al*., 2002). To reconstruct the regulatory events involved, co‐expression analyses revealed a highly ordered and hierarchical genetic network. Several lines of evidence suggested that *WRKY40* directly regulates the connected hub genes described here. Our qRT‐PCR analysis indicated that *WRKY40* was up‐regulated in both the HR and ZS cultivars, with significantly higher transcription in HR than in ZS. Notably, it was dramatically down‐regulated in VIGS *MPK3* cotton lines suggesting that *WRKY40* may be downstream of the MPK signal pathway. Plant WRKYs are involved in plant pathogen interactions (Pandey and Somssich, 2009). In *Arabidopsis thaliana*, a mutant lacking the *WRKY40* repressor results in exaggerated expression of some defense genes, such as enhanced disease susceptibility1 (*EDS1*), AP2‐type transcription factor, and JA‐related genes (Pandey *et al*., 2010). WRKYs are activated by MAPK cascades, such as *MPK3* and *MPK6;* both were up‐regulated after whitefly infestation, which is in accordance with previous reports (Kim and Zhang, 2004; Skibbe *et al*., 2008). The majority of transcription factors identified in this report were suppressed after whitefly infestation, which is similar to *V. dahliae* infection suggesting that down‐regulation of some gene networks might be required to modulate the plant immune signalling network (Xu *et al*., 2011).

### Phytohormone signalling pathways are involved in insect resistance

Whitefly infestation also induced the expression of genes associated with phytohormone signalling pathways (Artico *et al*., 2014). Phytohormones such as JA, ET, SA and GA are thought to play critical roles in complex signalling cascades and orchestrate the induction of insect defenses (Bari and Jones, 2009; Sun *et al*., 2011). In this report, JA‐related genes (*LOX*,* JAZ*,* FAD*,* AOC4*) were down‐regulated at all time points in the ZS cultivar. Similarly, *AOC4*, which is involved in the JA synthesis pathway, is a major candidate gene for *BPH* infestation of rice (Wang *et al*., 2012). Moreover, microarrays indicated that a large portion of wounding‐ and herbivory‐induced responses are mediated by the JA pathway (Kempema *et al*., 2007; Reymond *et al*., 2004). Our results further showed the involvement of GA in defense responses to whiteflies. Our qRT‐PCR data showed that *BGL1* was up‐regulated after whitefly infestation. In our previous report, over expression of *BGL1* resulted in elevated free GA content in the transplastomic plant and caused an increase in *N*. *tabacum* trichome density, which may protect the plants from aphids and whiteflies (Jin *et al*., 2011). Mechanical wounding and insects activate MPK both locally and systemically in tomato and *N. attenuate* (Wu *et al*., 2007). MAPKs are also required for the accumulation of defensive metabolites (Hettenhausen *et al*., 2014). In this report, silencing *MPK3* and *MPK6* by VIGS revealed that both kinases regulated the gene expression of WRKYs as well as genes in the JA and ET pathways, which highlights their central role in whitefly resistance. In summary, the complexity underlying expression of these genes illustrates the extraordinary interconnectedness of the signalling pathways regulated by phytohormones in response to whitefly infestation.

### Stress responses possibly interact with secondary flavonoid metabolites

Secondary metabolites that are derived from multiple pathways, including terpenoids, flavonoids, phenylpropanoids and aromatic compounds, play fundamental roles in the plant response to invading pathogens and insect infestations. Despite causing little substantive damage, aphids and whiteflies elicit remarkable changes in secondary metabolism (Walling, 2000). Interestingly, flavonoid metabolite‐related genes, such as *PAL* (phenylalanine ammonia‐lyase), *TPS*,* F3H*,* DFR* and *CHS*, were down‐regulated during the later stage of whitefly infestation (48 h). Our qRT‐PCR confirmed the down‐regulation of *CHS* and *F3H*. Compared to previous reports, the comprehensiveness of our study expands the limited molecular basis underlying cotton responses to whiteflies (Dubey *et al*., 2013; Morant *et al*., 2008). Previous reports on cotton resistance to *V. dahliae* demonstrated a rapid accumulation of flavonoids, which were measured after pathogen inoculation in both control and inoculated cotton roots, which suggested the possibility of flavonoids acting as signal molecules (Xu *et al*., 2011). Divol reported that several genes involved in sugar remobilization in celery, including glutamine synthase, were induced by aphid infestation (Divol *et al*., 2005). Our results also confirmed that starch and sucrose metabolism‐related genes (glutamine synthase, alpha‐amylase‐like, glycosyl hydrolase, *PGL1* and UDP‐glucosyltransferase) were up‐regulated in both the HR and ZS cultivars.

## Experimental procedures

### Three‐year greenhouse study to evaluate resistance of multiple cotton cultivars to whitefly infestation

Three greenhouse experiments were conducted at the National Key Laboratory of Crop Genetic Improvement at Huazhong Agricultural University, Wuhan City, Hubei, China. Experiment 1 was initiated in 2012 (April 10–June 10), Experiment 2 in 2013 (April 15–June 20) and Experiment 3 in 2014 (May 10–July 12). More than 400 cotton cultivars (*G. hirsutum*), each replicated five times, were used in each experimental setting. Seeds were directly sown into autoclaved nutrient soil in 12‐inch pots. The plants were irrigated according to accepted greenhouse horticultural practices. Weeds were controlled by hand and cultivation. For artificial whitefly infestation, tobacco plants (*N. tabacum* L.), which had previously been infested with ~300–400 adult whiteflies (*B. tabaci* type B) per plant were placed during the four‐leaf stage of cotton between the cotton plots and shaken to encourage movement of whitefly adults to the cotton plants. In this setup, ~40 cotton plants were exposed to a single whitefly‐infested tobacco plant. After exposure for 1 day, the tobacco plants were returned to separate glasshouse rooms to restore the whitefly populations. Artificial infestation using the infested tobacco plants was repeated once a week for 3 weeks to insure transfer to cotton.

The whitefly population in each experiment was assessed at the 10‐leaf stage of cotton plants (~7 weeks old). The number of adult whiteflies on the cotton plants was manually determined by careful inspection of the underside of leaves. The number of eggs and nymphs on each leaf were determined using a binocular microscope with all whitefly developmental stages imaged using a stereoscopic microscope (LEICA, MZFLIII). Trichomes on the fully expanded leaves (i.e. fourth leaf from the top) were recorded as above with three microscope fields including one control leaf and three leaves from either the whitefly resistant cotton cultivar (HR) or the whitefly susceptible cultivar (ZS).

For gossypol content analysis, full‐expanded leaves (the fourth leaf from the top) were dried in vacuum freeze‐dryer. The freeze‐dried leaves were crushed using zirconia beads for 1.5 min at 30 Hz with a Mixer Mill (MM 400, Retsch). For each sample, 100 mg powder was weighed and extracted by ultrasound in a mixture of acetonitrile and water (65 : 35 V/V). Samples were further processed by vibration, centrifugation and suction filter filtration prior to HPLC analysis (Agilent Technologies, 1260 Infinity). HPLC separation was performed using an Agilent TC C‐18 (150 mm × 4.6 mm, 5 μm) column at 25 °C and a mobile phase consisting of acetonitrile and 0.2% phosphoric acid (85 : 15 V/V) under a flow rate of 1.0 mL/min. UV detection was set at 238 nm and the injected sample volume was 20 μL. Analyses were replicated three times for the HR and ZS cultivars.

### Whitefly infestation of HR/ZS cultivars in the small container

*Gossypium hirsutum* (cv. HR and ZS cultivars) seeds were sown for germination in 1/2 MS medium. After germination, the seeds were maintained in the dark for 2 days, plantlets were transferred to the light house for 5 days at 28 ± 2 °C (day/night) with relative humidity of 50%–60% and a 16 h day/8 h light photoperiod until the plantlets developed flat cotyledons. Whiteflies (*B. tabaci* type B) were reared on potted cotton plants in the greenhouse at 28 ± 2 °C and 70% relative humidity. Freshly emerged and adult whiteflies were collected from the greenhouse by aspiration into falcon tubes. Fifty adult whiteflies were collected and released onto plantlets with two flat cotyledons grown in a small box (Figure S1). Adult whiteflies were removed from the cotton plant after 12, 24 and 48 h infestation. Control plants (control) were grown as the others but without whitefly infestation. After whitefly infestation, two leaves from these plantlets were immediately frozen in liquid nitrogen and stored at −70 °C until use. All experiments were performed in three biological replicates.

### RNA isolation and sequencing

Twenty‐four samples from the HR and ZS cultivars were used for RNA sequencing. Total RNA was isolated from each sample using a modified guanidine thiocyanate method described previously (Tu *et al*., 2007). Around 0.4 μg of total RNA was used for library construction and sequencing on an Illumina Genome Analyzer (Illumina, San Diego, CA, USA) at the National Key Laboratory of Crop Genetic Improvement at Huazhong Agricultural University (Wuhan, China). Prior to library construction, an Agilent 2100 Bioanalyzer (Agilent, CA, USA) was used to confirm the quality and quantity of RNA such that the rRNA ratio (28 s/18 s) was >1.5 and the RNA integrity number >7. In summary, 0.1–0.4 μg total mRNA was purified and fragmented using PCR plates with a magnetic plate stand. Fragmented mRNA was reverse‐transcribed to cDNA using random primers and Superscript II (Invitrogen, Carlsbad, CA). Blunt‐ended cDNA was generated by end repair and then ligated to yield 3′ adenine base overhangs. Oligonucleotide adapters with thymine overhangs were ligated to the cDNA and added to the adapter index for each library. The library fragments were enriched by PCR amplification and 1 003 930 166 raw ~100 bp pair‐end reads were generated on an Illumina HiSeqTM 2000. The RNA‐seq data are available from NCBI of sequence Read Archive (SRA project: PRJNA286935).

### Trimming and mapping of reads

Illumina sequence data were selectively filtered using SolexaQA2.2 (Cox *et al*., 2010) to remove read lengths <30 bp and lacking phred quality scores <20 at each nucleotide. Clean reads (~90% of the total read count) were mapped to the *G. hirsutum* genome, and a recent internal cotton transcriptome using Tophat with default parameters (Trapnell *et al*., 2010). Mapped reads were normalized using the DESeq Bioconductor package (Anders and Huber, 2010). Data quality analysis was conducted by visualizing results of principal component analysis (PCA).

### Identification and functional annotation of DEGs

The mapped sequenced reads for all of the identified genes were used for differential expression analysis by the DESeq method with a *P* 0.01 (Anders and Huber, 2010). Gene expression levels were calculated as reads per kilobase of transcript sequence per million base pairs sequenced (FPKM) using Cufflinks and Cuffdiff (Trapnell *et al*., 2012). Based on these statistical analyses, genes with *P *<* *0.01 and an absolute value |log2 ratio ≥ 2 were considered to be significant differentially expressed genes (DEGs). The final DEGs were identified by DESeq and cuffdiff. DEGs were initially annotated using BlastX (*e*‐value < 10^−5^) with the *Arabidopsis thaliana* TAIR10 protein database and then the NCBI non‐redundant protein database. GO enrichment analysis was conducted using Blast2GO (https://www.blast2go.com/) with FDR < 0.05. The DEGs were clustered based on their expression patterns by the Genesis K‐means method (Sturn *et al*., 2002). GO terms were analysed for enrichment in a test group compared to a reference group using Fisher's Exact Test with FDR (Benjamini and Hochberg, 1995). All of the DEGs were also subjected to KOBAS 2.0 analysis (http://kobas.cbi.pku.edu.cn/home.do) and significant pathways were selected at a *P *<* *0.05 (Wu *et al*., 2006). DEGs were compared to KEGG orthology and the *Arabidopsis* pathway. These DEGs were also compared with NCBI public data generated from cotton plants infested with chewing or sap‐sucking insects.

### Data integration and network construction

A total of 16 RNA‐Seq datasets were generated with an Illunina Hiseq 2000, which comprised transcriptomic profiling of ZS and HR cultivars after whitefly infestation at four time points. Weighted gene co‐expression network (WGCNA) analysis was performed as described previously (Langfelder and Horvath, 2008; Zhang and Horvath, 2005). The R scripts and tutorials are available at (http://labs.genetics.ucla.edu/horvath/CoexpressionNetwork/Rpackages/WGCNA/). The datasets for 3720 DEGs from the ZS cultivar were clustered according to their log2 (FPKM) values. Initially, the absolute value of the Pearson's correlation coefficient was calculated for all pairs of genes in these datasets. We then raised these correlations to a soft‐threshold power (ß = 8) to approximate scale‐free topology within the network. From these scaled correlations, topological overlaps, which summarize the degree of shared connections between any two genes, were calculated for all genes. A value of 0.85 was selected to differentiate branches of the dendrogram using the dynamic tree‐cutting algorithm (Zhang and Horvath, 2005), resulting in a network containing 15 modules. The different colours represent different modules, genes not represented by a module are shown in grey scale. The association of module eigengenes and the gene expression were determined as follows: for each module, the expression of module genes was depicted using a heatmap. The module GO terms were tested by Fisher's exact test. Different thresholds were subsequently selected for exporting the gene networks with VisANT visualization software (Hu *et al*., 2013).

### RT‐PCR and qRT‐PCR

Candidate transcriptomic genes were validated by qRT‐PCR analysis using three biological replicates. Gene‐specific primers (Table S7) were designed using Primer 5.0 and synthesized commercially (Genscript Bioscience, Nanjing, China). First‐strand cDNA was synthesized from 3 μg of total RNA using SuperScript III Reverse Transcriptase (Invitrogen) in accordance with the manufacturer's instructions and reverse transcribed into cDNA followed by 50× dilution. The RT‐PCR program was as follows: one cycle of 5 min at 95 °C as an initial denaturation step followed by denaturation for 30 s at 95 °C, annealing for 30 s at 60 °C, extension for 30 s at 72 °C for 35 cycles and a final step at 72 °C for 10 min in a 20 μL volume. qRT‐PCR was performed in a 20 μL reaction volume containing 9.6 μL of 10× diluted cDNA as the template, 0.2 μL of each 10 μm forward and reverse gene‐specific primer, and 10 μL of SsoFast EvaGreen Supermix With Low ROX (Bio‐Rad, Hercules, CA). The qRT‐PCR reactions were performed on an Applied Biosystem 7500 real‐time PCR system (Applied Biosystem, Foster City, CA) for 2 min at 95 °C, followed by 40 cycles for 5 s at 95 °C and 35 s at 60 °C. The qRT‐PCR product sizes ranged from 80 bp to 200 bp. Relative quantification of gene expression was calculated and normalized using *GhUBQ7* (GenBank accession number: DQ116441) as an internal standard (Tu *et al*., 2007). After qRT‐PCR, the dissociation curve was used to confirm the specificity of the primers. Dissociation parameters were 95 °C for 15 s, 60 °C for 1 min and 95 °C for 15 s. The comparative Ct (2^−ΔΔCt^) method was used to calculate the fold‐changes in gene expression level (Pfaffl, 2001).

### Virus‐induced gene silencing (VIGS) in cotton followed by whitefly infestation

To investigate the putative function of several candidate insect resistant genes, VIGS was performed using the *GhMPK6*,* GhMPK3*,* GhHSP20* and *GbCLA* (*cloroplastos alterados 1*) genes with the resulting silenced plants subsequently challenged with whitefly infestation. The VIGS experiments were performed according to our previous reports (Gao *et al*., 2013; Xu *et al*., 2014) with minor modifications. The *Agrobacterium tumefaciens* harbouring tobacco rattle virus (TRV) vectors were prepared according to Fradin's description (Fradin *et al*., 2009). Target gene fragments for *TRV:GhMPK3*,* TRV:GhMPK6*,* TRV:GhHSP* and *TRV:GhCLA1* (positive control) were amplified from the *G. hirsutum* genome to generate TRV vectors (PCR primers are shown in Table S7). Digested PCR fragments were then ligated into the *TRV:00* plasmid (Liu *et al*., 2002). The resulting vectors were transformed into *A. tumefaciens* GV3101 by electroporation. Agro‐infiltration was performed using a needle‐less syringe on 10‐day‐old seedlings for *G. hirsutum* cv. HR, ZS, and *G. barbadence* cv.7124(Whitefly resistant cultivar). The inoculated seedlings were then transferred to a controlled environmental chamber and grown at 25 °C in the dark for 2 days and then exposed to light (16 h/8 h light/dark photoperiod cycle) for 1 week.

As shown in Fig 10, the bleaching leaf phenotype was observed 10 days after infiltration with *TRV:GbCLA1* plants, which suggested the VIGS system was successful. At this stage, *TRV:GhMPK3*,* TRV:GhMPK6*,* TRV:GhHSP* and *TRV:00* plants were divided into two groups: one group was subjected to whitefly infestation in the greenhouse and the other group was maintained in a controlled environmental chamber. Whitefly infestation was carried out for 2 weeks using whiteflies that originated from wild tobacco plants. Expression levels of the VIGS target genes were evaluated in VIGS and *TRV:00* (CK) plants by RT‐PCR and qRT‐PCR as described above.

## Author contributions

S.X.J., X.L.Z., H.D. conceived and designed the project and revised the manuscript. J.Y.L. analysed transcriptome data, performed expression analysis experiment and wrote this manuscript. L.Z.Z. contributed data in Figure 1 and performed whitefly resistant test in cotton. J.J.H. interpreted transcriptome data and revised the manuscript. S.J.L. performed VIGS experiment.

## Conflict of interest

All the authors have declared no conflict of interest.

## Supporting information


**Figure S1** Cotton whitefly infestation within a sealed chamber.
**Figure S2** Comprehensive evaluation of the RNA‐Seq data. (a, b) Analysis of all samples was performed based on FPKM data using PCA. (c) All samples were clustered using the *pvclust* package.
**Figure S3** Test for differential expression in cotton before and after whitefly infestation. Scatter plot indicates the log2 fold change versus mean. Red indicates DEGs detected with a *P *<* *0.01 and a multiple testing adjustment.
**Figure S4** Ven diagram showing the overlap of DEGs detected using different analysis methods.
**Figure S5** Pie charts represent GO terms (biological_process level 2) in the various treatment groups.
**Figure S6** Network construction of related results. (a) Analysis of network topology through different soft‐thresholding limits. (b) Ten modules DEGs were showed by a hierarchical clustering dendrogram. (c) Clustering of the modules. (d) The heatmap shows the eigengene adjacency.
**Figure S7** Correlation between qRT‐PCR and RNA‐Seq results.

**Table S1** FPKM values for functionally annotated DEGs at different time points after whitefly infestation. FPKMs were calculated using cufflinks and biological replicates were merged with cuffdiff.
**Table S2** List of all DEGs detected by RNA‐Seq analysis after whitefly infestation in the HR and ZS cultivars compared with control cotton.
**Table S3** Expression pattern of DEGs common to both HR and ZS cultivars following hierarchical clustering.
**Table S4** Gene ontology enrichment analysis of all DEGs at FDR < 0.05.
**Table S5** KEGG enrichment analysis of all DEGs at FDR < 0.05.
**Table S6** Detailed list of resistance‐related genes in the HR and ZS cultivars that are induced or suppressed in response to whitefly infestation.
**Table S7** List of primers used for qRT‐PCR.
