# Supplementary material for: Transcriptome analysis reveals a comprehensive insect resistance response mechanism in cotton to infestation by the phloem feeding insect Bemisia tabaci (whitefly)
Source: Plant Biotechnol J. 2016 Mar 31;14(10):1956–75. doi: 10.1111/pbi.12554 (PMC5042180; doi:10.1111/pbi.12554)
Supplement: Supplementary file 1 — Figure S1 Cotton whitefly infestation within a sealed chamber. Figure S2 Comprehensive evaluation of the RNA‐Seq data. (a, b) Analysis of all samples was performed based on FPKM data using PCA. (c) All samples were clustered using the pvclust package. Figure S3 Test for differential expression in cotton before and after whitefly infestation. Scatter plot indicates the log2 fold change versus mean. Red indicates DEGs detected with a P < 0.01 and a multiple testing adjustment. Figure S4 Ven diagram showing the overlap of DEGs detected using different analysis methods. Figure S5 Pie charts represent GO terms (biological_process level 2) in the various treatment groups. Figure S6 Network construction of related results. (a) Analysis of network topology through different soft‐thresholding limits. (b) Ten modules DEGs were showed by a hierarchical clustering dendrogram. (c) Clustering of the modules. (d) The heatmap shows the eigengene adjacency. Figure S7 Correlation between qRT‐PCR and RNA‐Seq results. [file PBI-14-1956-s002.pptx]

## Slide 1
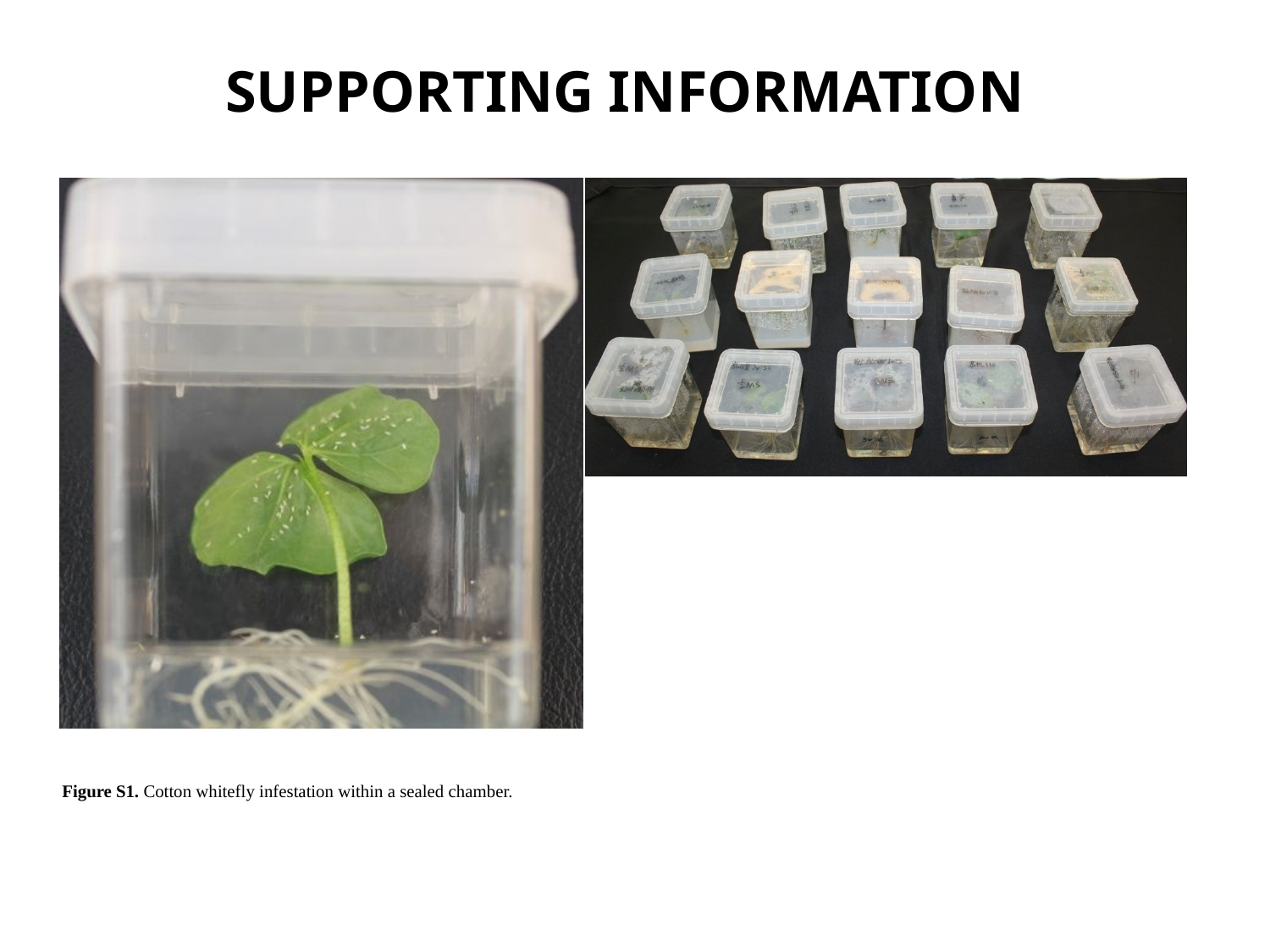

SUPPORTING INFORMATION
Figure S1. Cotton whitefly infestation within a sealed chamber.

## Slide 2
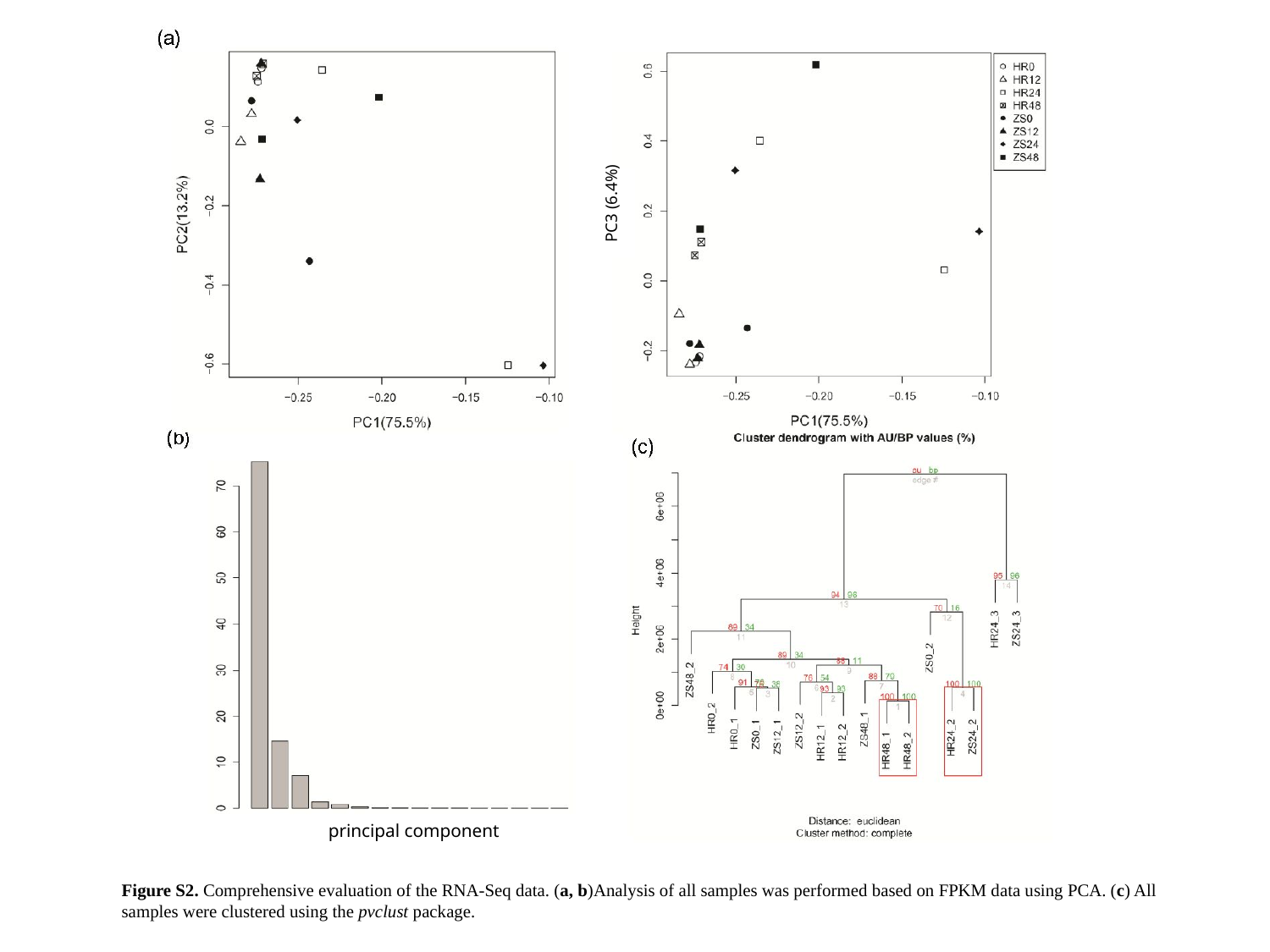

PC3 (6.4%)
principal component
Figure S2. Comprehensive evaluation of the RNA-Seq data. (a, b)Analysis of all samples was performed based on FPKM data using PCA. (c) All samples were clustered using the pvclust package.

## Slide 3
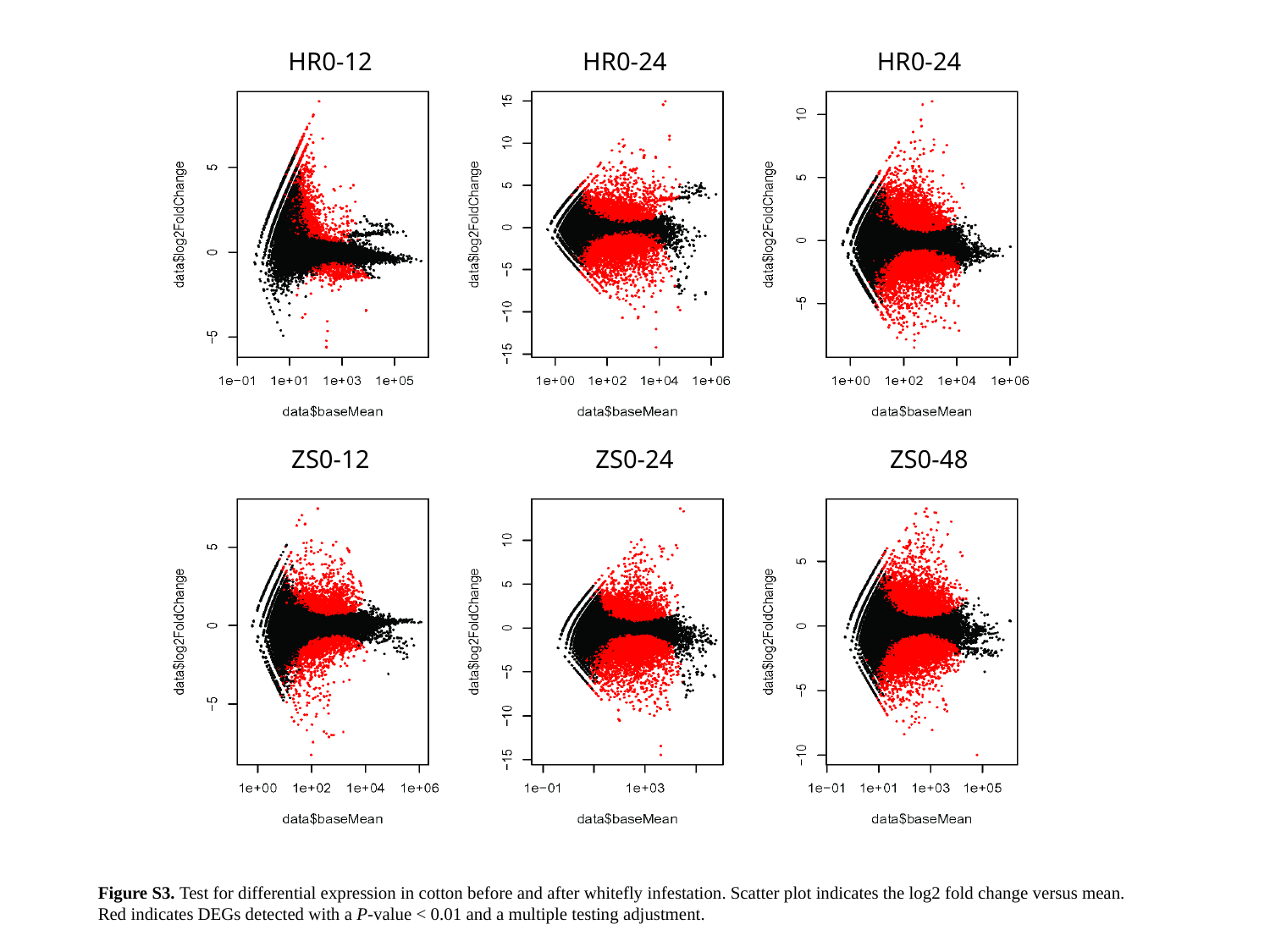

HR0-12
HR0-24
HR0-24
ZS0-12
ZS0-24
ZS0-48
Figure S3. Test for differential expression in cotton before and after whitefly infestation. Scatter plot indicates the log2 fold change versus mean.
Red indicates DEGs detected with a P-value < 0.01 and a multiple testing adjustment.

## Slide 4
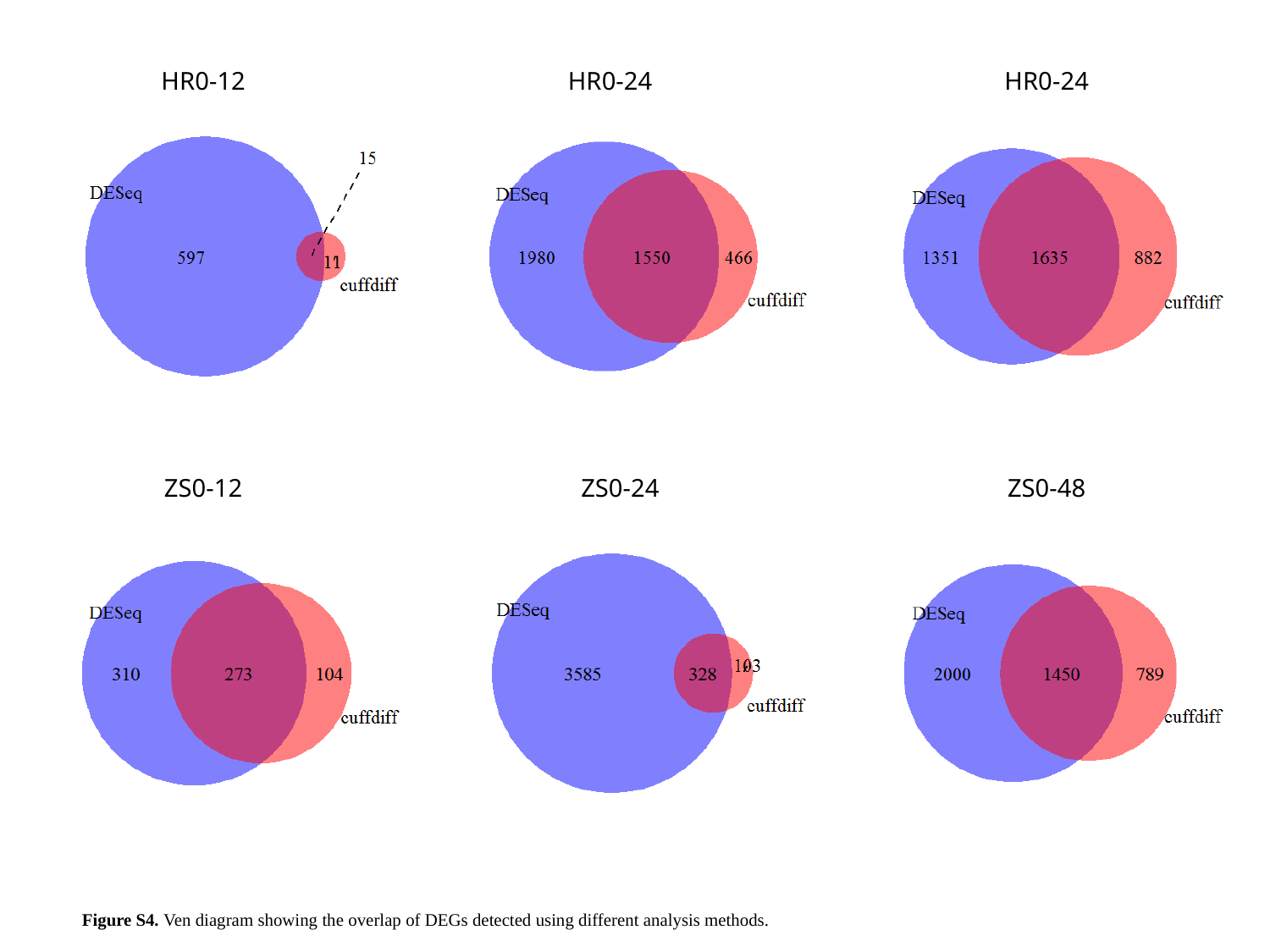

HR0-12
HR0-24
HR0-24
ZS0-24
ZS0-48
ZS0-12
Figure S4. Ven diagram showing the overlap of DEGs detected using different analysis methods.

## Slide 5
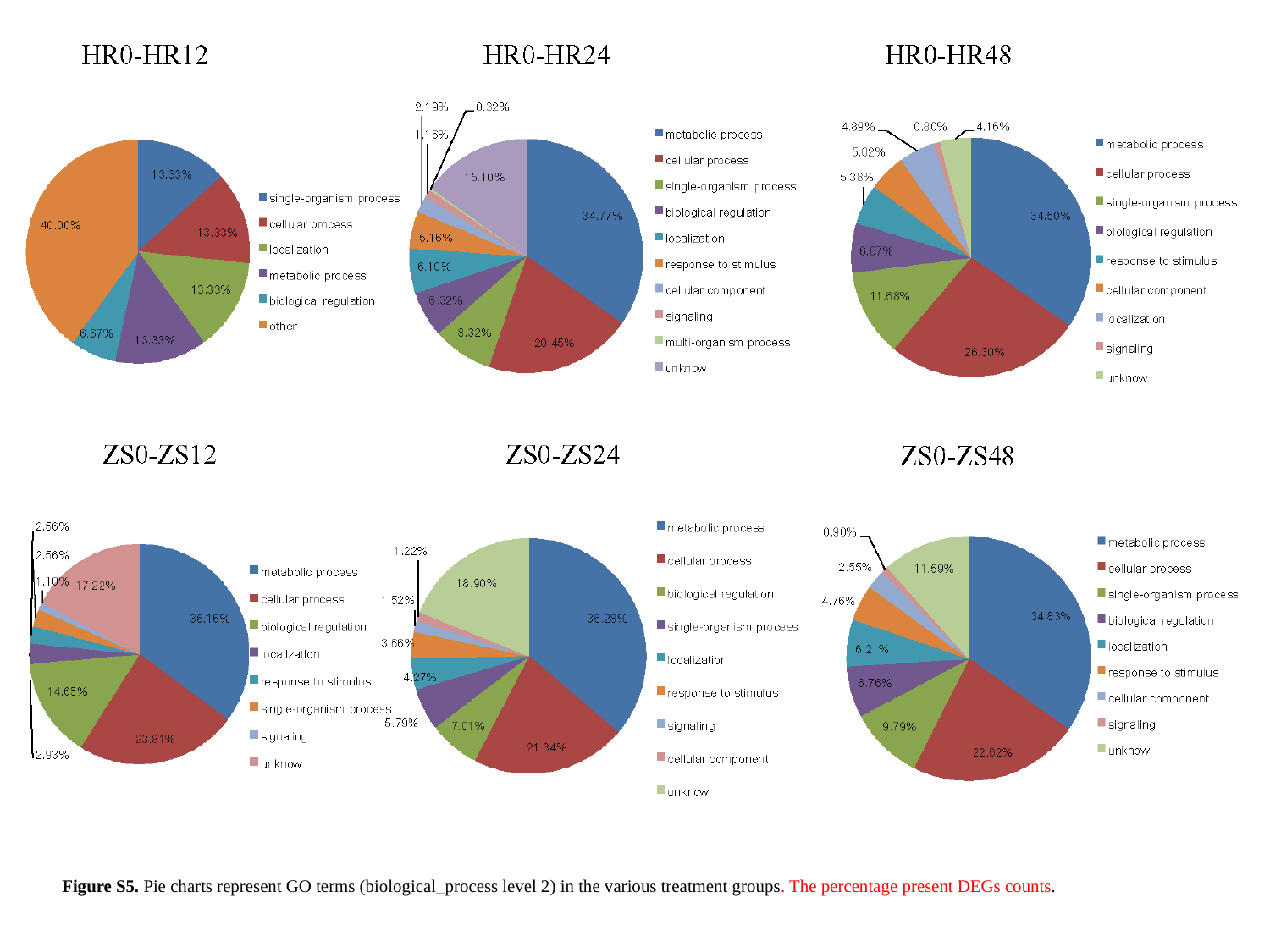

Figure S5. Pie charts represent GO terms (biological_process level 2) in the various treatment groups. The percentage present DEGs counts.

## Slide 6
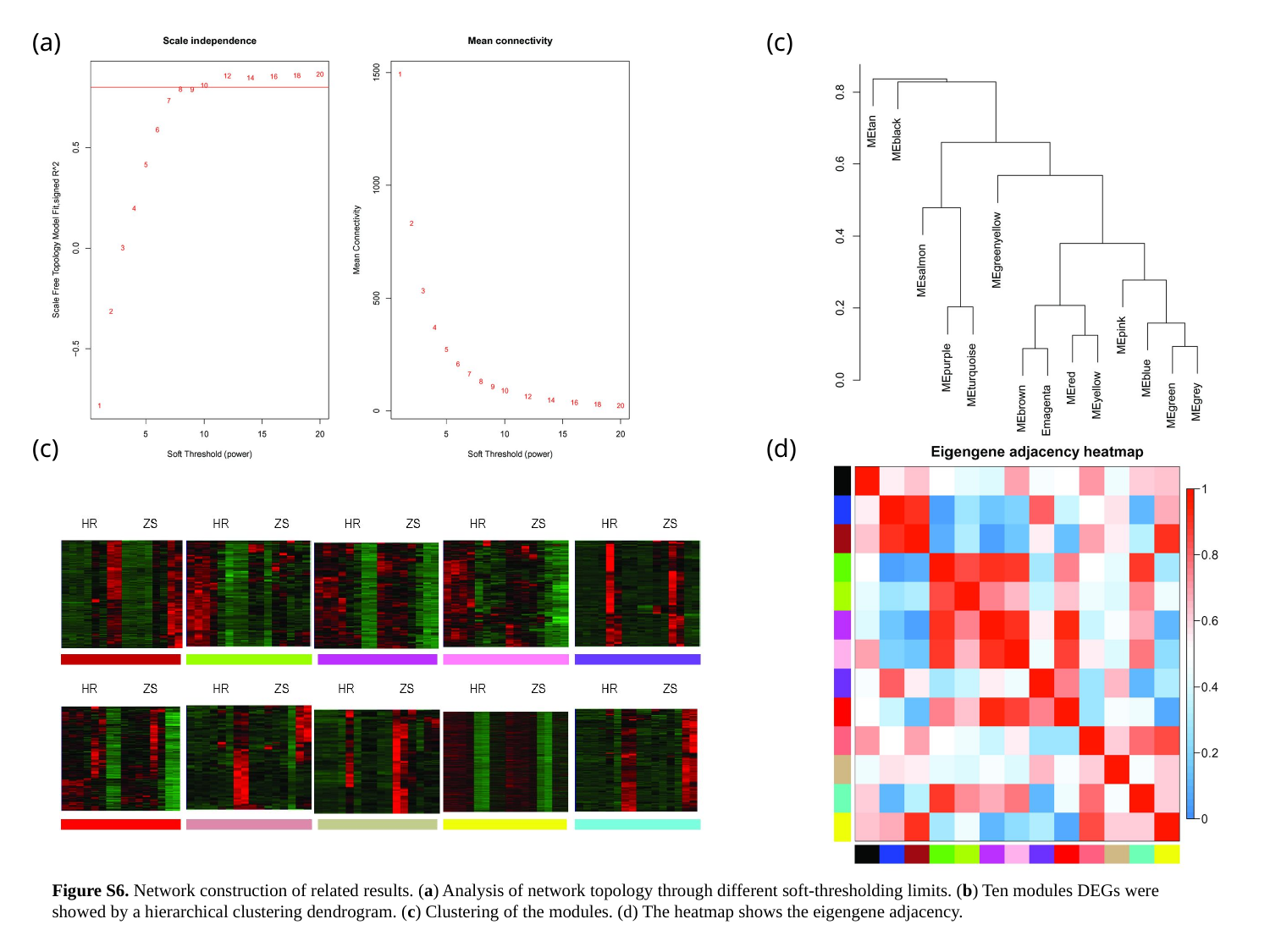

(a)
(c)
(c)
(d)
Figure S6. Network construction of related results. (a) Analysis of network topology through different soft-thresholding limits. (b) Ten modules DEGs were showed by a hierarchical clustering dendrogram. (c) Clustering of the modules. (d) The heatmap shows the eigengene adjacency.

## Slide 7
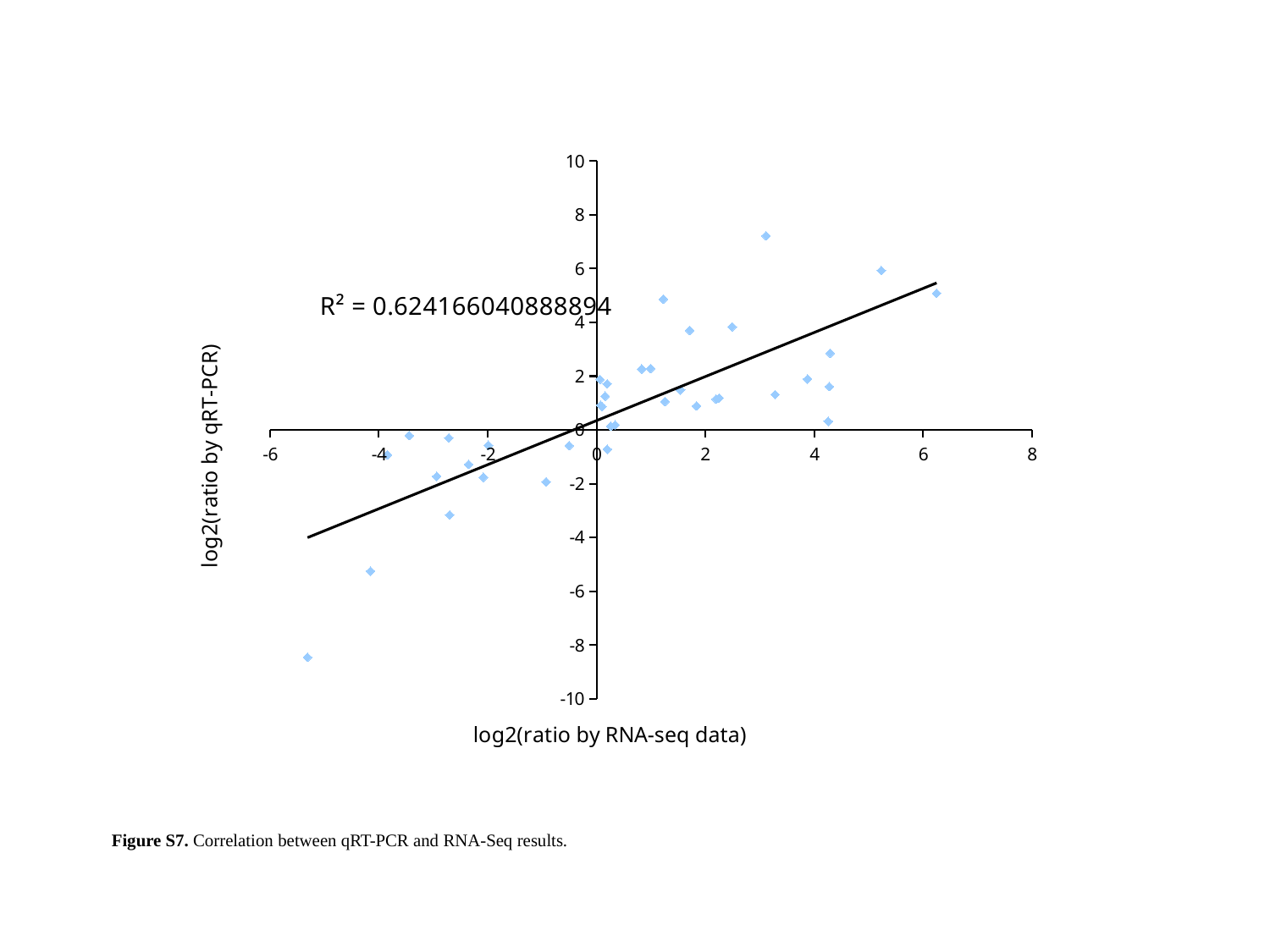

### Chart
| Category | |
|---|---|Figure S7. Correlation between qRT-PCR and RNA-Seq results.
